# Potential Distribution Prediction and Metabolite Analysis of *Clematis tangutica* *(Maxim.) Korsh*. On the Qinghai Plateau

**DOI:** 10.1002/ece3.72110

**Published:** 2025-09-30

**Authors:** Haiwang Zhang, Xiaozhu Guo, Xiaoqiang Wei, Lihui Wang, Qiwen Zhong, Xuemei Sun

**Affiliations:** ^1^ Qinghai University Xining China; ^2^ Academy of Agriculture and Forestry Sciences, Qinghai University Xining China; ^3^ Lanzhou University Lanzhou China; ^4^ Laboratory for Research and Utilization of Qinghai Tibet Plateau Germplasm Resources Xining China

**Keywords:** *C. tangutica*, habitat distribution, MaxEnt, Qinghai plateau, secondary metabolites

## Abstract

*Clematis tangutica(Maxim.) Korsh*. is an ornamental and medicinal plant endemic to the Central and East Asian plateaus. To comprehensively understand the natural habitat distribution and potential medicinal value of *C. tangutica* on the Qinghai Plateau, this study employed the MaxEnt model to predict its potential habitat distribution on the Qinghai Plateau and assess the impacts of climate change. Leaf metabolites from five different altitudes were analyzed using UPLC‐MS/MS to explore variations in metabolite accumulation. The results showed that the environmental variables exerting the greatest influence on the potential suitable distribution of *C. tangutica* were UV‐B seasonality (uvb2, percent contribution: 44.4%) and elevation (elev, percent contribution: 28.9%). High and moderately suitable habitats were mainly distributed in eastern, central, and southern Qinghai, covering a total of 299,300 km^2^. Under future climate change scenarios, the total suitable habitat area is expected to decrease, with more significant reductions under high‐emission pathways. Metabolomic analysis identified 1362 metabolites, including flavonoids, amino acids and derivatives, phenolic acids, alkaloids, and terpenoids, with the highest accumulation occurring at altitudes between 2500 and 3500 m. Notably, key bioactive compounds, such as flavonoids and phenolic acids, were enriched at higher elevations, suggesting a strong link between environmental stress and secondary metabolite biosynthesis. These findings provide new insights into the ecological adaptability and medicinal potential of *C. tangutica* , highlighting its role as a model species for understanding plant‐environment interactions in extreme habitats.

## Graphical abstract

This study provides a comprehensive analysis of *Clematis tangutica* 's habitat distribution and metabolite composition under varying environmental conditions. The MaxEnt model identified UV‐B seasonality and elevation as the primary factors shaping its distribution, with the most suitable habitats concentrated in central and eastern Qinghai. Metabolomic analysis revealed significant altitude‐dependent variations in bioactive compounds, with peak accumulation observed between 2500 and 3500 m.

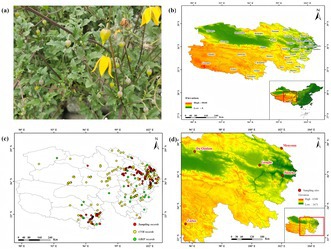

## 1 Introduction

**FIGURE 1:**
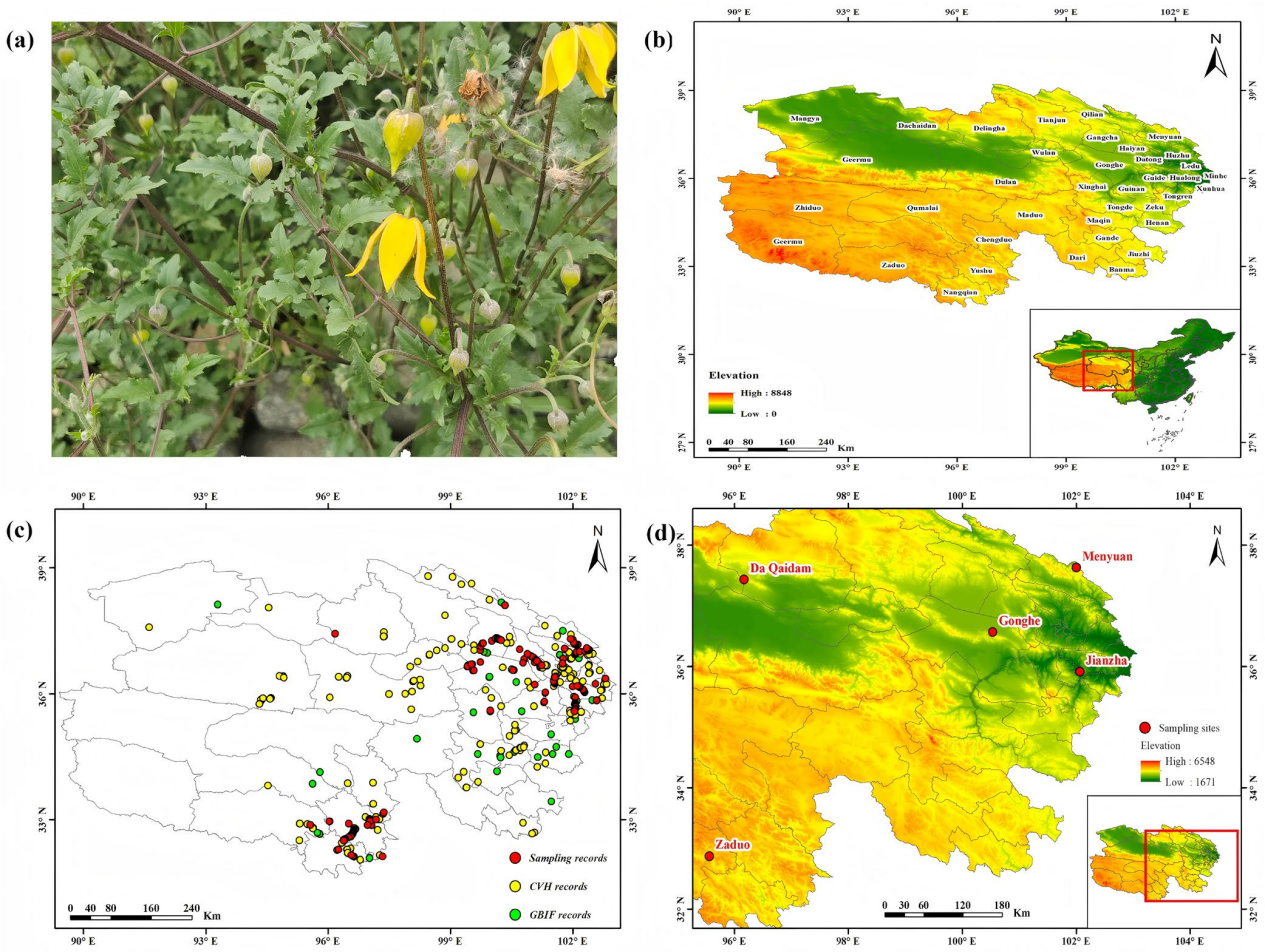
Distribution records and sampling locations for metabolite analysis of *C. tangutica* on the Qinghai Plateau. (a) *C. tangutica*. (b) Geographic location of the Qinghai–Tibet Plateau. (c) Distribution records of *C. tangutica* . (d) Sampling locations for metabolomics analysis.

*Clematis tangutica(Maxim.) Korsh*. is a perennial deciduous vine in the Ranunculaceae family and Clematis genus, primarily found in the alpine meadows, shrubs, and rocky areas of China, Kazakhstan, Kyrgyzstan, India, and Nepal (Figure 1a). In China, it thrives in the high‐altitude regions of Qinghai, Tibet, western Xinjiang, western Sichuan, and southeastern Gansu. Known for its ecological adaptability, *C. tangutica* exhibits resistance to drought, cold, and radiation (Guo et al. 2024; Wei et al. 2023b). The yellow flowers of *C. tangutica*, with purple edges, distinguish it from other Clematis species. The flowers are fragrant and bloom for a long period, making them suitable for exhibition cut flowers and vertical greening materials, thus having ornamental value (Guo, Wang et al. 2023). However, *C. tangutica's* habitat in high‐altitude areas with harsh conditions difficult, necessitating the development of artificial cultivation techniques (Qu 2017; Zhao et al. 2023). *C. tangutica* can also be used medicinally in its entirety. In Tibetan medicine, it promotes blood circulation, removes blood stasis, and treats indigestion. The plant effectively treats cardiovascular and cerebrovascular diseases and has medicinal effects such as expelling pus, healing sores, and dissolving lumps (Chen et al. 2023; Zhao et al. 2016). Secondary metabolites such as flavonoids, phenolic acids, alkaloids, terpenes, and quinones are the main active components. For instance, flavonoids inhibit tumor cell growth and induce apoptosis (Yang, Zhang, et al. 2023). Saponins, particularly the abundant triterpenoid saponins found in *C. tangutica* , serve as characteristic chemical markers. They exhibit bioactivities including anticancer, antibacterial, antifungal, anti‐inflammatory, and cardioprotective effects (Moghimipour et al. 2014). Triterpene saponins are the main active saponins in *C. tangutica* (Wei et al. 2023a; Zhao et al. 2016). The content of key secondary metabolites in medicinal plants correlates with the suitability of their habitats. In general, highly suitable habitats offer more favorable environmental conditions for the accumulation of key secondary metabolites, which may in turn lead to differences in medicinal efficacy (Li et al. 2020; Zhan et al. 2022). Therefore, evaluating the adaptability of *C. tangutica* under different environmental conditions and quantitatively analyzing changes in its main active substances are prerequisites for the rational development and utilization of its medicinal value.

Global climate change and environmental degradation pose serious threats to sustainable plant utilization, leading to significant habitat shrinkage, migration, changes in species dominance, survival, turnover, and community structure (Bernatchez et al. 2024; Briscoe et al. 2023; Carlson et al. 2022). Using species distribution models (SDMs) to predict species distribution changes has become a common approach in many climate change studies (Lee‐Yaw et al. 2022; Lu et al. 2024). Among numerous ecological niche models, the Maximum Entropy model (MaxEnt) stands out in current species distribution research due to its small sample requirements, ease of operation, strong applicability, and excellent predictive performance and stability (Elith et al. 2011; Phillips and Dudík 2008). MaxEnt can predict the potential impacts of climate change on species' habitats, assess the relationship between species distribution and environmental variables (Shi et al. 2023; Zhao, Cui, et al. 2021), identify habitats for rare and endangered species (Yang et al. 2021), predict potential distribution areas for invasive species (Zhang, Song, et al. 2021), and forecast the spread of pests and diseases (Hwang et al. 2022; Recopuerto‐Medina et al. 2024). During prediction, the choice of environmental variables directly influences both the accuracy and ecological interpretability of the model. Variables with high resolution, strong ecological relevance, and low collinearity better define the species' ecological niche boundaries, improving prediction reliability. In contrast, redundant or overly abstract variables may lead to model overfitting or obscure ecological insights (Mod et al. 2016). Moreover, temporal and spatial dynamics of variables, such as climate and soil changes, impact the adaptability and transferability of predictions (Thorson et al. 2023). Therefore, variable selection and validation should consider species' physiological and ecological traits alongside actual distribution data. This approach ensures the model is both statistically robust and ecologically meaningful.

Although a previous study preliminarily examined the relationship between the ecological niche of *C. tangutica* and environmental factors, indicating that the species is mainly distributed in the eastern Qinghai–Tibet Plateau, with the most suitable habitats concentrated in western Sichuan Province (Yuan et al. 2024), there remains a lack of systematic potential habitat prediction using MaxEnt and assessments under future climate scenarios. Building on this earlier research, the present study applies the MaxEnt model to predict the potential habitat distribution of *C. tangutica* on the Qinghai Plateau and further integrates ultra‐performance liquid chromatography–tandem mass spectrometry (UPLC–MS/MS) to investigate the enrichment patterns of key secondary metabolites along altitudinal gradients, thereby elucidating the ecological adaptation mechanisms linking habitat suitability to medicinal value. The former quantitatively assesses the influence of climatic and environmental factors on the habitat suitability of *C. tangutica* , revealing potential distribution patterns and habitat shifts under future climate scenarios. The latter employs metabolomics to analyze the accumulation patterns of functional bioactive compounds in *C. tangutica* across various environmental gradients. We aim to explore the potential ecological adaptation relationship between habitat suitability and secondary metabolite profile changes in *C. tangutica* , verifying whether highly suitable habitats promote the enrichment of functional bioactive compounds. This approach seeks to reveal the coupling mechanism between environmental adaptability and medicinal value, providing integrative evidence from ecology and chemical biology to support the conservation and sustainable utilization of *C. tangutica* . It should be noted that this study has limitations, including the assumption that environmental factors such as soil and ultraviolet radiation remain constant in future climate projections, and a limited metabolomic sample size covering only major altitudinal gradients, which may constrain the extrapolation of results.

## 2 Materials and Methods

### 2.1 Study Area

The Qinghai Plateau, situated in the northeastern Qinghai‐Tibet Plateau, known as the “Roof of the World,” spans longitudes 89°35′ to 103°04′ E and latitudes 31°36′ to 39°19′ N (Figure 1b). This region is characterized by extensive alpine cold deserts and thin air, resulting in Qinghai's distinctive plateau climate of high altitude, low temperatures, aridity, and strong radiation. More than 80% of Qinghai lies above 3000 m in elevation, with annual average temperatures ranging from −5.8°C to 8.6°C. Temperatures vary with altitude. The average annual precipitation is approximately 300 mm, with most areas receiving less than 400 mm, and the driest regions receiving under 20 mm annually. The region experiences high radiation intensity, with annual total radiation ranging from 586 to 741 kJ/cm^2^, and long sunshine duration, between 2000 and 3600 h per year. Qinghai is rich in plant resources, with a diverse mix of phytogeographical elements that contribute to its abundant plant diversity. It is a central area for the concentrated distribution of unique alpine and polar plants in China and one of the most floristically diverse alpine regions globally. Nearly 12,000 species of higher angiosperms and over 800 species of ferns are found here, including 75 families, 331 genera, and over 1000 species of economic value, such as medicinal, fiber, starch, oil, and ornamental plants. Representative endemic alpine species on the plateau include *Ophiocordyceps sinensis* (an ascomycetous fungus), *Lycium ruthenicum*, *Pulveroboletus ravenelii*, and *Potentilla anserina* . These species thrive under the plateau's unique environmental conditions and contain distinct bioactive compounds with specific effects. Therefore, it is crucial to prioritize and implement conservation and management strategies for these wild plant resources. (Sources: The People's Government of Qinghai Province Website, http://www.qinghai.gov.cn/).

### 2.2 Distribution Record Collection and Selection

A total of 291 global distribution records of *C. tangutica* were collected from the Global Biodiversity Information Facility (GBIF, https://www.gbif.org/) and the Chinese Virtual Herbarium (CVH, http://cvh.ac.cn/) (Zhang et al. 2023) Additionally, 260 occurrence points of wild *C. tangutica* were collected through field surveys. To minimize errors from clustering effects in subsequent modeling, ENMTools (http://purl.oclc.org/enmtools) was used to filter and select these distribution records by removing duplicates and invalid points (Warren et al. 2021), ensuring only one record per 10 km^2^ grid. This process resulted in 130 valid sample points. Although sampling points were filtered at a 10 km^2^ scale, environmental variables were rasterized at a 30″ (approximately 1 km) resolution for prediction to balance model robustness with spatial precision. Finally, ArcGIS 10.6.1 (Esri, Redlands, California, USA) was used to map the distribution records of *C. tangutica* on the Qinghai–Tibet Plateau (Figure 1c).

### 2.3 Environment Variables Preparation and Selection

Nineteen bioclimatic variables from the World Climate Database (http://www.worldclim.org/) were selected for modeling the distribution of *C. tangutica* . Elevation data were also sourced from this database, while slope and aspect were calculated using ArcGIS 10.6.1 (Amiri et al. 2020). Soil data were obtained from the global soil organic matter dataset of the National Tibetan Plateau Data Center (http://data.tpdc.ac.cn), which includes 18 topsoil variables with a thickness of 0–30 cm. UV data were obtained from the global UVB radiation dataset of the gIUV database (http://www.ufz.de/gluv/). Prior to model construction, all environmental variables were standardized to a consistent spatial resolution, coordinate system, and boundaries (He et al. 2023). The spatial resolution was set to 30″ (approximately 1 km), the coordinate system was WGS1984, and the boundaries were 90° N, −90° S, 180° E, and −180°W. To ensure the ecological relevance of background points, all environmental layers were cropped before modeling to the Qinghai Plateau (31°–39° N, 89°–103° E), which covers *C. tangutica* known distribution. Background points in the MaxEnt model were randomly generated solely within this cropped area, rather than across the global extent, to avoid mismatches between background environments and the species' ecological niche.

For future climate projections, we selected the latest Coupled Model Intercomparison Project 6 (CMIP6) model from the IPCC Sixth Assessment Report (AR6) of 2021 (Eyring et al. 2016). We used the Beijing Climate Center's medium‐resolution climate system model (BCC‐CSM2‐MR) to analyze the periods 2041–2060 (2050s) and 2061–2080 (2070s) under the SSP2‐4.5 and SSP5‐8.5 scenarios in the WorldClim database. The BCC‐CSM2‐MR model is widely used for simulating the global climate's response to increased greenhouse gas concentrations (Wu et al. 2020; Xiao‐Ge et al. 2019). Notably, we assumed that data on topography, soil, and UV intensity would remain unchanged in the future during model construction.

Before constructing the model, environmental variables were imported into the MaxEnt model and run with default parameters. Environmental variable values at sample presence points were extracted using ArcGIS 10.6.1, and Pearson correlation analysis was performed with R 4.1.3 (Li et al. 2024). In the preliminary MaxEnt model run, environmental variables were ranked by contribution as follows: elev, uvb2, bio18, bio1, uvb4, bio15, bio19, uvb3, bio4, t_bs, bio7, bio9, uvb5, uvb1, aspect, bio17, slope, usda, awc, t_clay, bio11, t_cec, bio14, t_texture, teb, bio8, t_ece, and t_esp; the remaining variables contributed less than 1%. Pearson correlation analysis was performed on environmental variable values extracted at species occurrence points. For bioclimatic variables with |*r*| ≥ 0.8, those with higher contributions were retained for the final MaxEnt model. Ultimately, the selected variables were bio1, bio4, bio8, bio9, bio11, bio17, bio18, bio19, elev, usda, t_clay, t_bs, t_esp, uvb1, uvb2, uvb3, uvb4, and uvb5 (Table S1, Figure S1).

### 2.4 Model Optimization and Simulation

Although most researchers tend to use default parameters when running the MaxEnt model, models built with these parameters can be relatively complex and may suffer from overfitting, making the prediction results challenging to interpret. To optimize the MaxEnt model, adjustments were made to the feature combination (FC) and regularization multiplier (RM) parameters (Zhao, Deng, et al. 2021). In this study, the “block” method was employed to divide the 130 *C. tangutica* records into 4 equal parts, with 3 parts used for training and 1 part for testing. The range of RM was set from 0.5 to 4 with an increment of 0.5, resulting in a total of 8 RM parameters. Regarding the FC parameters, six feature combinations were utilized: L, LQ, H, LQH, LQHP, and LQHPT. These combinations were tested using the ENMeval package in R 4.1.3, resulting in 48 parameter combinations (Kass et al. 2021; Muscarella et al. 2014). The model's fitting and complexity were evaluated using the Akaike information criterion (AICc) (Portet 2020). Finally, the model parameter combination with the smallest AICc value was selected as the optimal predictive model parameters (RM = 1, FC = LQHPT) (Figure S2a).

The ‘asc’ format files of significant environmental variables and the final distribution records of *C. tangutica* were imported into the MaxEnt model. Feature classes (FC) were set to LQHPT, and the Jackknife method was used to calculate the contribution rates and permutation importance of environmental variables. Settings were configured to create response curves with the following specifications: output format set to ‘Logistic’, output file type to ‘asc’, random test percentage to 25%, regularization multiplier to 1, and replicates to 10 with ‘Crossvalidate’ as the run type (Guo, Zhang, et al. 2023). All other parameters were kept at their default values, and the run was initiated after finalizing the settings. After the run, subsequent analysis was performed using ArcGIS 10.6.1 and the SDM toolbox.

Model accuracy is evaluated using threshold‐independent Receiver Operating Characteristic (ROC) curves and the area under the ROC curve (AUC). Data were divided into test and training sets during the calculation process. Generally, a higher AUC for the training set than for the testing set suggests a good model fit; however, a substantially higher training AUC may indicate overfitting. A higher AUC value for training data compared to test data indicates good predictive performance (He et al. 2023). When repeated 10 times, the software calculates the average AUC value, which ranges from 0 to 1.0. An AUC value between 0 and 0.5 indicates poor model performance; 0.6–0.9 indicates moderate performance; and above 0.9 indicates highly accurate predictions.

### 2.5 Sampling and Metabolite Analysis

#### 2.5.1 Sampling

From June to August in 2022 and 2023, we surveyed wild *C. tangutica* populations across the Qinghai Plateau and collected flowering‐stage leaves from five locations: Jianzha (JZ; E102.0635132, N35.9119252; 2060 m), Menyuan (MY; E102.002984, N37.628280; 2641 m), Gonghe (GH; E100.535315, N36.564461; 3191 m), Da Qaidam (DQ; E96.165820, N37.431948; 3590 m), and Zaduo (ZD; E95.558919, N32.868805; 4252 m). Samples were immediately frozen in liquid nitrogen and stored at −80°C. Each location included three biological replicates for metabolomic analysis (Figure 1d).

#### 2.5.2 Metabolite Extraction and Profiling Using UPLC‐MS/MS Techniques

Leaf samples of *C. tangutica* from five regions were sent to Metware Biotechnology Co. Ltd. (Wuhan, China) for metabolomic analysis. Metabolites were first extracted and analyzed using ultra‐high‐performance liquid chromatography–tandem mass spectrometry (UPLC‐MS/MS). In the experiment, 50 mg of frozen, ground sample powder was dissolved in 1.2 mL of 70% methanol. The solution was vortexed thoroughly and centrifuged at 12,000 rpm for 3 min to obtain the supernatant. The supernatant was filtered through a 0.22 μm membrane and used for UPLC‐MS/MS analysis (Romera et al. 2018).

Liquid chromatography was performed using an Agilent SB‐C18 column (1.8 μm, 2.1 mm × 100 mm). Mobile phase A was ultrapure water with 0.1% formic acid, and mobile phase B was acetonitrile with 0.1% formic acid. The elution gradient was as follows: the proportion of phase B started at 5%, linearly increased to 95% over 9 min, and was held for 1 min. Then, from 10 to 11.1 min, the proportion of phase B was decreased back to 5%, and the system was equilibrated at 14 min (Yang et al. 2024). The flow rate was set to 0.35 mL/min, the column temperature was maintained at 40°C, and the injection volume was 2 μL.

Mass spectrometry detection was conducted in electrospray ionization (ESI) mode. The ESI source temperature was set to 550°C, with ion spray voltages of 5500 V in positive ion mode and −4500 V in negative ion mode. The pressures of ion source gas I, gas II, and curtain gas (CUR) were set to 50 psi, 60 psi, and 25 psi, respectively. The collision‐induced dissociation (CID) parameters were set to high intensity (Kusano et al. 2015). Mass spectrometer calibration was conducted in triple quadrupole (QQQ) mode with a 10 μmol/L polypropylene glycol solution, and in linear ion trap (LIT) mode with a 100 μmol/L standard solution (Liu et al. 2023). The QQQ scanning mode used multiple reaction monitoring (MRM), with the collision gas (nitrogen) flow rate set to medium intensity. The declustering potential (DP) and collision energy (CE) for each MRM ion pair were optimized to monitor specific ion pairs for each elution phase.

#### 2.5.3 Statistical Analysis

The qualitative analysis of metabolites was based on secondary spectral information from the Metware Database (MWDB), while quantitative analysis was performed in MRM mode using MultiaQuant software for chromatographic peak integration and correction. Metabolites were detected using UPLC‐MS/MS. Differential metabolites were then screened based on orthogonal partial least squares discriminant analysis (OPLS‐DA) results, combined with variable importance in projection (VIP) values and fold change. The data were log‐transformed (log_2_) before OPLS‐DA analysis. A 200‐time permutation test was then conducted to avoid overfitting. Metabolites with a fold change ≥ 2 or ≤ 0.5 and a VIP value ≥ 1 were considered significant differential metabolites (Chen et al. 2013). Principal component analysis (PCA) was conducted using the prcomp function in R 4.1.3, with data scaled to unit variance before the analysis. Identified metabolites were annotated using the KEGG compound database (http://www.kegg.jp/kegg/compound/) and mapped to the KEGG pathway database (http://www.kegg.jp/kegg/pathway.html). Bubble plots were analyzed using MetaboAnalyst. Statistical analysis of all data was performed using Excel 2019.

## 3 Results

### 3.1 The MaxEnt Model Predicts the Potential Distribution of *C. tangutica* 's Habitat and Changes in Area

#### 3.1.1 Evaluation of Model Accuracy and Analysis of Main Environmental Variables

Using MaxEnt to analyze the potential distribution of *C. tangutica* under current climate conditions, the results showed that the AUC values were 0.992, indicating excellent model prediction accuracy (Figure S1). Among the 18 environmental variables, UV‐B seasonality (uvb2), elevation (elev), and precipitation of coldest quarter (bio19) had the greatest impact on the potential distribution of *C. tangutica* , with contribution rates of 44.4%, 28.9%, and 8.2%, respectively, and a cumulative contribution rate of 81.5%. The contribution rates of other environmental variables did not exceed 5% (Table 1). The top‐ranked single environmental variables for training gain were UV‐B seasonality (uvb2), elevation (elev), Mean UV‐B of Highest Month (uvb3), and Sum of Monthly Mean UV‐B during Highest Quarter (uvb5), indicating these variables contributed more effective information related to the distribution of *C. tangutica* and had a greater impact on its distribution (Figure S3). Combining the percentage contribution rates and jackknife test results, uvb2 and elev are the main environmental factors affecting the distribution of *C. tangutica* under current climate conditions. UV‐B and elevation variables have the most significant impact, whereas precipitation, temperature, and soil variables have less influence on the distribution of *C. tangutica's* suitable habitat.

**TABLE 1:** Percent contribution and permutation importance of environmental variables under current climatic conditions.

Variable	Percent contribution	Permutation importance
uvb2	44.4	13.2
elev	28.9	7.3
bio19	8.2	5
bio4	4.2	17
uvb4	4.2	21.8
bio9	3	1.8
bio18	2.9	10.1
bio11	1.7	0.1
bio8	1.5	2.1
bio1	0.3	17.2
bio17	0.3	1
uvb3	0.1	0.1
uvb5	0.1	1.5
uvb1	0.1	0.3
usda	0	0.5
t_clay	0	0.8
t‐bs	0	0
t_esp	0	0

**FIGURE 2:**
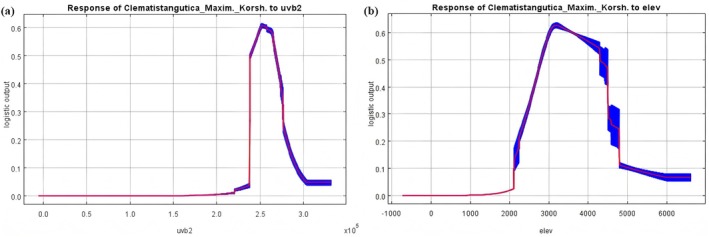
Probability response curves of the main environmental variables affecting the distribution of *C. tangutica* . (a) Response curve of uvb2; (b) Response curve of elev.

To further clarify the biological tolerance and habitat preference of *C. tangutica* , the potential suitable distribution area was predicted based on current climatic conditions. Response curves of the two main environmental variables that significantly affect the distribution of *C. tangutica* were analyzed (Figure 2a,b). The critical values of the main environmental variables (existence probability > 0.5) were obtained: uvb2 ranged from 239,062.77 J·m^−2^·d^−1^ ~ 268,462.59 J·m^−2^·d^−1^, and elev ranged from 2801.29 to 4292.36 m. The suitability of *C. tangutica* reached its maximum when uvb2 was between 250,890.28 J·m^−2^·d^−1^ ~ 257,648.86 J·m^−2^·d^−1^ (existence probability > 0.60), and when elev was between 3095.09 m and 3300.76 m (existence probability > 0.62).

#### 3.1.2 Predicted Distribution and Area of Suitable Habitats for *C. tangutica* Under Current Climatic Conditions

**FIGURE 3:**
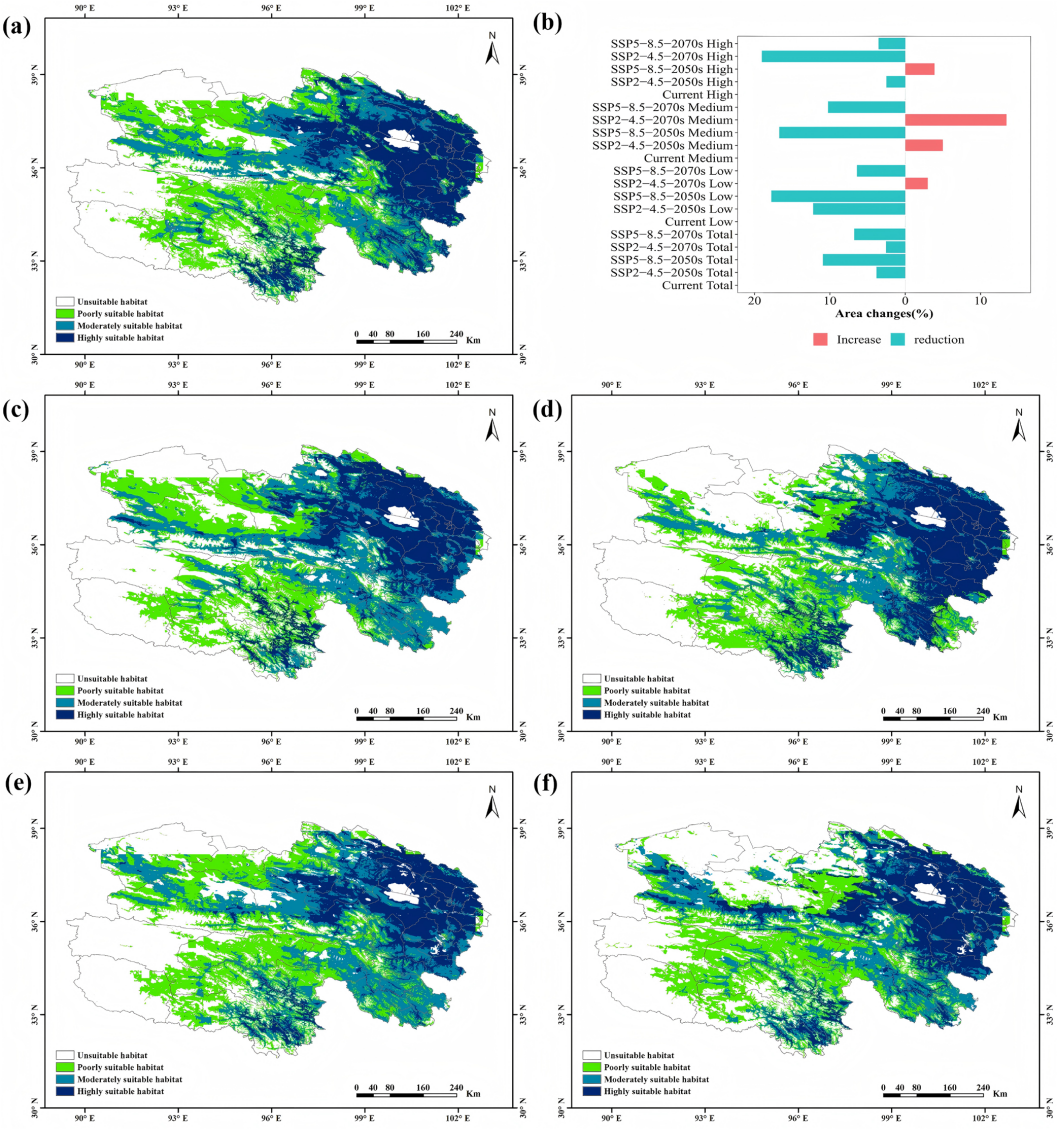
Based on the MaxEnt model predicting the current and future potential distribution and area changes of *C. tangutica* . (a) Distribution of suitable habitats under current climate; (b) Changes in habitat area; (c) Distribution of suitable habitats under SSP2‐4.5‐2050s scenario; (d) Distribution of suitable habitats under SSP5‐8.5‐2050s scenario; (e) Distribution of suitable habitats under SSP2‐4.5‐2070s scenario; (f) Distribution of suitable habitats under SSP5‐8.5‐2070s scenario.

Based on the MaxEnt model prediction results, the potential suitable habitat for *C. tangutica* in the Qinghai Plateau under current climatic conditions was visualized and classified using Arcmap 10.6.1 software and the Reclassify tool (Figure 3a). Dark blue areas represent highly suitable habitats, blue areas represent moderately suitable habitats, green areas represent low suitability habitats, and white areas represent unsuitable habitats. The highly suitable habitats for *C. tangutica* are mainly distributed in eastern, central‐northern, and some southern parts of Qinghai, covering an area of approximately 145,000 km^2^, which accounts for about 21.01% of the total area (approximately 690,000 km^2^). Moderately suitable habitats are mostly around the highly suitable areas, mainly distributed in central, western, and some southern parts, covering an area of approximately 154,300 km^2^, which accounts for about 22.36% of the total area. Low suitability habitats are mainly distributed in the western, central‐southern, and sporadic eastern regions, covering an area of approximately 183,700 km^2^, which accounts for about 26.62% of the total area. Additionally, other regions are unsuitable for *C. tangutica* , covering an area of approximately 173,800 km^2^, which accounts for about 25.19% of the total area.

#### 3.1.3 Future Climate Scenarios for Predicting the Distribution and Area Changes of *C. tangutica*

Based on the same criteria, we predicted the potential suitable habitats of *C. tangutica* for 2040–2060 (2050s) and 2060–2080 (2070s) using the MaxEnt model under SSP2‐4.5 and SSP5‐8.5 scenarios. We obtained the potential suitable habitat distribution maps of *C. tangutica* under future climate scenarios (Figure 3c–f), and the changes in the area of each suitability zone (Figure 3b, Table S2).

According to the climate scenarios for the 2050s and 2070s, the location and area of each suitability zone for *C. tangutica* changed to varying degrees. In the 2050s, the highly suitable zone area decreased by 2.51% under the SSP2‐4.5 pathway and increased by 3.89% under the SSP5‐8.5 pathway. The moderately suitable zone area increased by 4.99% under the SSP2‐4.5 pathway and decreased by 16.71% under the SSP5‐8.5. The low suitability zone area decreased under both pathways by 12.21% and 17.77%, respectively. The total suitable area decreased under both pathways, with greater reductions observed with increased radiative forcing. In the 2070s, the highly suitable zone area decreased by 19.02% under the SSP2‐4.5 and 3.55% under the SSP5‐8.5. The moderately suitable zone area increased by 13.44% under the SSP2‐4.5 and decreased by 10.24% under the SSP5‐8.5. The low suitability zone area increased by 2.99% under the SSP2‐4.5 and decreased by 6.41% under the SSP5‐8.5. Consistent with the 2050s, the total suitable area decreased under both pathways in the 2070s, with greater reductions observed with increased radiative forcing.

### 3.2 Metabolite Analysis

**FIGURE 4:**
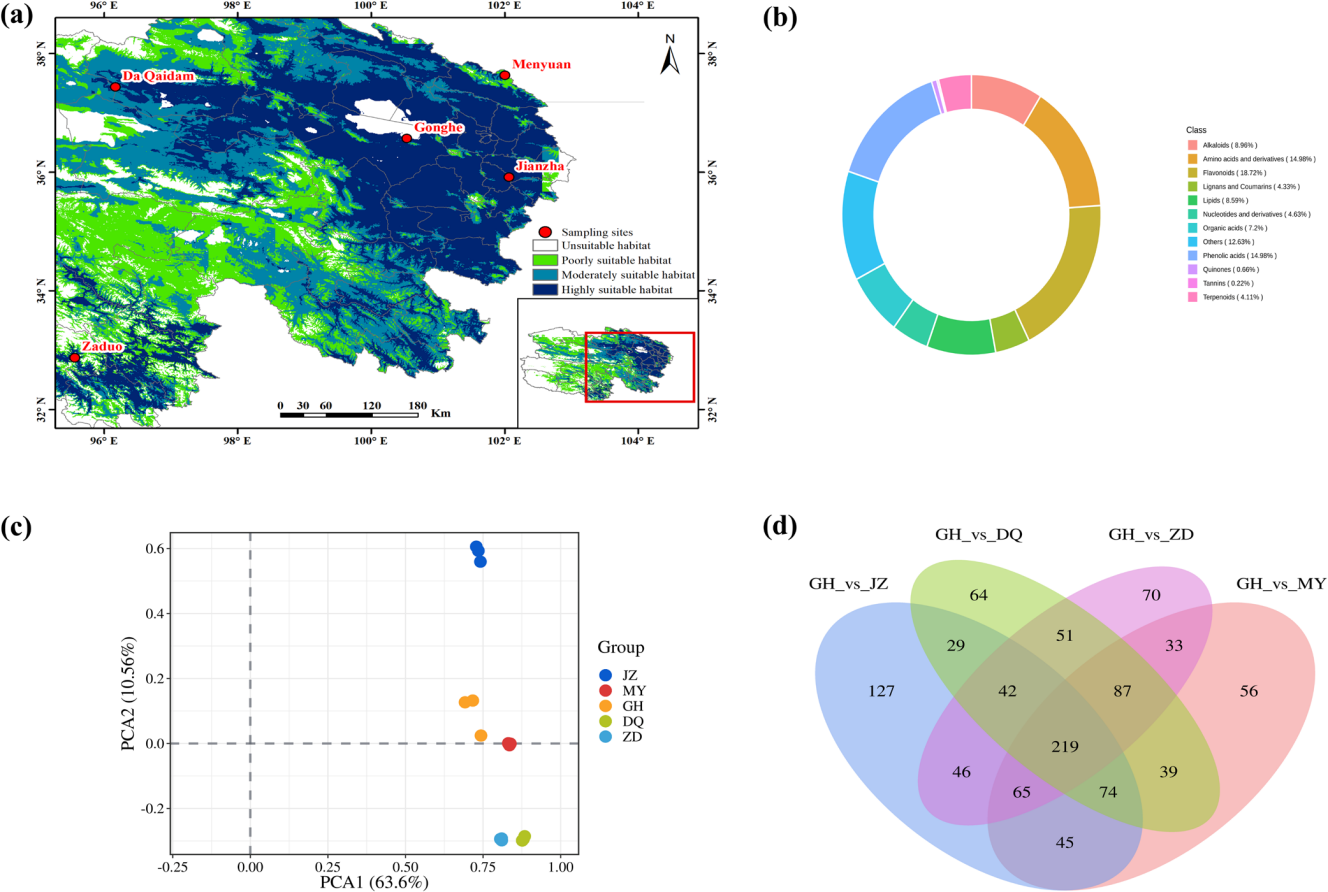
Distribution of suitable areas for sampling sites and metabolomic analysis of *C. tangutica* leaves. (a) Distribution of suitable areas for sampling sites; (b) Composition and content proportion of major metabolites; (c) Principal component analysis; (d) Venn diagram of differential metabolites.

The MaxEnt model predictions indicate that UV‐B seasonality and elevation are the main environmental variables influencing the distribution of *C. tangutica* . Given the close relationship between UV intensity and elevation, it raises the question: Does altitude also affect the accumulation of metabolites in *C. tangutica* ? To clarify the main metabolites and differences in *C. tangutica* leaves at different altitudes, we collected leaf samples from five regions at different altitudes. According to the model predictions, sampling points JZ, MY, GH, and ZD are in high suitability habitats, while DQ is in a moderate suitable habitat (Figure 4a).

Based on the Human Metabolome Database (HMDB), Kyoto Encyclopedia of Genes and Genomes (KEGG), and multiple reaction monitoring (MRM), we conducted a qualitative and quantitative analysis of metabolites in *C. tangutica* leaves from the five sampling points. We detected a total of 1362 metabolites by UPLC‐MS/MS, including flavonoids (255, 18.72%), amino acids and derivatives (204, 14.98%), phenolic acids (204, 14.98%), alkaloids (122, 8.96%), lipids (117, 8.59%), organic acids (98, 7.2%), nucleotides and derivatives (63, 4.63%), lignans and coumarins (59, 4.33%), terpenoids (56, 4.11%), quinones (9, 0.66%), tannins (3, 0.22%), and others (172, 12.63%) (Figure 4b).

#### 3.2.1 PCA and OPLS‐DA

Based on PCA analysis, the grouping and intra‐group variation of *C. tangutica* samples from five altitudes were analyzed. The results show that two principal components (PCA1 and PCA2) explain 63.6% and 10.56% of the variance in the original dataset, respectively. The five samples are clearly separated, with each sample's three biological replicates clustering closely together. Along PCA1, samples from relatively lower altitudes (JZ, MY, GH) are separated from those at higher altitudes (DQ and ZD). Additionally, on PCA2, sample JZ exhibits significant metabolic differences from other groups, while MY and GH show similar metabolic profiles, and DQ and ZD have closer metabolic profiles, indicating significant differences among *C. tangutica* samples from different altitudes (Figure 4c). According to the above predictions, the optimal altitude range affecting the potential distribution of *C. tangutica* is 3095.09 to 3300.76 m (existence probability > 0.62). Therefore, we selected GH (altitude 3191 m) as the core group for differential metabolite analysis compared to other groups. OPLS‐DA analysis was used to screen variables contributing to differences among the five groups, with Q2 values exceeding 0.9 for all comparisons, indicating reliable models (Figure S4a–d). The S‐plot demonstrates significant differences in metabolites among samples from the five altitudes, illustrating altitude's impact on the accumulation of metabolic products in *C. tangutica* leaves (Figure S4e–h).

#### 3.2.2 Differential Metabolite and Enrichment Analysis

To further understand the differential metabolites in *C. tangutica* leaves at different altitudes, we used a combination of Variable Importance in Projection (VIP ≥ 1) and fold change (FC ≥ 2 or ≤ 0.5) to screen for differential metabolites, identifying a total of 1172 differential metabolites. The differential metabolites among the four comparison groups (GH vs. JZ/MY/DQ/ZD) were categorized into 12, 11, 12, and 11 categories, respectively. Specifically, 647 differential metabolites (379 up‐regulated, 268 down‐regulated) were identified between GH and JZ, 618 differential metabolites (334 up‐regulated, 284 down‐regulated) between GH and MY, 605 differential metabolites (239 up‐regulated, 366 down‐regulated) between GH and DQ, and 613 differential metabolites (207 up‐regulated, 406 down‐regulated) between GH and ZD. Additionally, a total of 219 differential metabolites were found across all four comparison groups, with 127 specific to GH versus JZ, 56 specific to GH versus MY, 64 specific to GH versus DQ, and 70 specific to GH versus ZD (Figure 4d).

**FIGURE 5:**
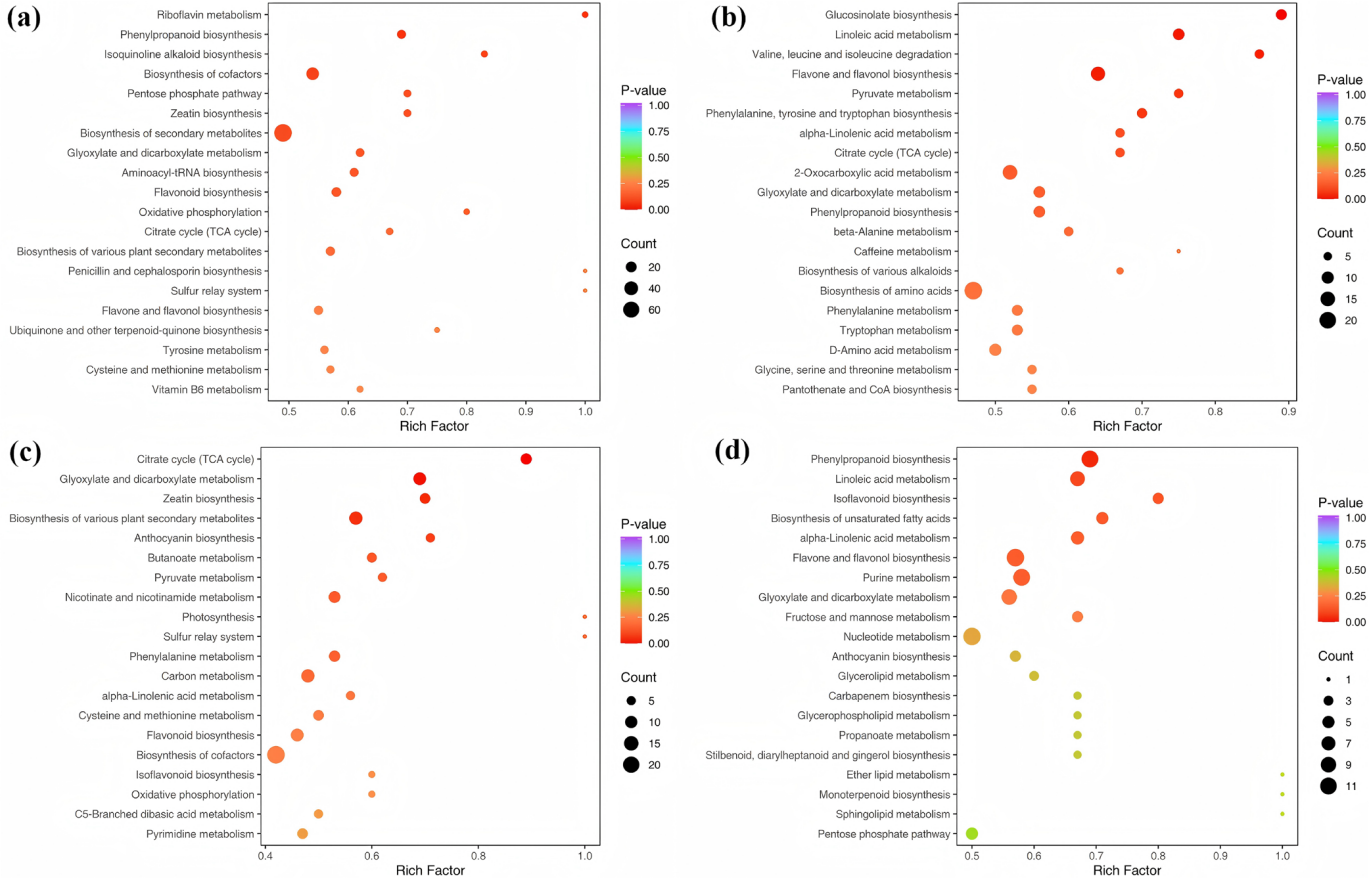
Bubble plot of KEGG pathway enrichment for differential metabolites. (a) KEGG enrichment of GH versus JZ; (b) KEGG enrichment of GH versus MY; (c) KEGG enrichment of GH versus DQ; (d) KEGG enrichment of GH versus ZD.

KEGG annotation and enrichment analysis of differential metabolites in each comparison group showed that the differential metabolites in GH and JZ, MY, DQ, and ZD involved 88, 82, 86, and 81 pathways, respectively. In GH vs. JZ, the significantly enriched pathways were riboflavin metabolism and phenylpropanoid biosynthesis (*p* < 0.05) (Figure 5a), indicating significant differences in the relative contents of vitamin B2, ketones, and phenylpropanoids in the leaves of *C. tangutica* between the GH and JZ regions. Metabolic pathways related to glucosinolate biosynthesis, linoleic acid metabolism, valine, leucine, and isoleucine degradation, flavone and flavonol biosynthesis, and pyruvate metabolism were significantly enriched in GH versus MY (*p* < 0.05) (Figure 5b), indicating significant differences in the relative contents of glucose, linoleic acid, valine, leucine, isoleucine, flavones, flavonols, and pyruvate between GH and MY. The significantly enriched metabolic pathways in GH vs. DQ were related to the citrate cycle (TCA cycle), glyoxylate and dicarboxylate metabolism, zeatin biosynthesis, and biosynthesis of various plant secondary metabolites (*p* < 0.05) (Figure 5c), indicating significant differences in the relative contents of citric acid, glyoxylic acid, dicarboxylic acids, zeatin, and other secondary metabolites between GH and DQ regions. Pathways related to phenylpropanoid biosynthesis were significantly enriched in GH versus ZD (*p* < 0.05) (Figure 5d), indicating significant differences in the relative contents of phenylpropanoids between GH and ZD. These results suggest that the differences in metabolite contents in *C. tangutica* leaves from the five different regions may be related to altitude.

#### 3.2.3 Flavonoids

A total of 255 flavonoid metabolites were identified, including 92 flavonols, 90 flavones, 22 flavanones, 15 anthocyanidins, 15 isoflavones, 7 chalcones, 7 flavanonols, and 6 flavanols. Among them, 6 flavones (chrysoeriol‐5‐O‐glucoside, luteolin‐7‐O‐(6″‐malonyl) glucoside, luteolin‐7‐O‐rutinoside, apigenin‐4′‐O‐glucoside, scutellarein‐7‐O‐glucuronosyl‐(1‐ > 2)‐ glucuronide, apigenin‐7‐O‐glucoside (cosmosiin)), 3 flavonols (quercetin‐3‐O‐galactoside (hyperin), kaempferol‐3‐O‐(6″‐O‐acetyl)glucoside, myricetin‐3‐O‐rutinoside), and 1 isoflavone (genistein‐7‐O‐galactoside) had the highest content, while the contents of flavanones, anthocyanidins, chalcones, flavanonols, and flavanols were relatively low. Some flavonoid compounds were also unique to specific altitudes, such as flavones (4′‐O‐glucosylvitexin), which were detected only in the GH, DQ, and ZD. This compound has been proven to have significant antioxidant activity and plays a role in treating hyperlipidemia, fatty liver, and heart protection (Kelebek 2016). Flavanones (pinocembrin‐7‐O‐neohesperidoside) were not detected in the ZJ group, and flavones (luteolin‐7‐O‐glucoside (cynaroside)) and flavonols (kaempferol‐3‐O‐galactoside (trifolin)) were not detected in the ZD. These substances were detected in the MY, GH, and DQ. These results indicate that the accumulation of flavonoid compounds in the flavonoid synthesis pathway is closely related to altitude.

The content of flavonoid compounds varies significantly at different altitudes, following a certain pattern. As altitude increases, the content of most flavonoid compounds first increases and then decreases, with the highest content found in the GH. For example, the content of flavones (chrysoeriol‐7‐O‐gentiobioside, luteolin (5,7,3′,4′‐Tetrahydroxyflavone)), flavonols (quercetin‐7‐O‐(6″‐malonyl) glucoside, 3,5,4′‐trihydroxy‐7‐methoxyflavone (rhamnocitrin)), anthocyanidins (peonidin‐3‐O‐glucoside, cyanidin‐3‐O‐glucoside (kuromanin)), isoflavones (2′‐hydroxygenistein, tectorigenin), and chalcones (dihydrocharcone‐4′‐O‐glucoside) first increase and then decrease with increasing altitude. Additionally, the content of a few flavonoid compounds decreases gradually with increasing altitude, such as flavones (chrysin‐7‐O‐glucoside), flavonols (kaempferol (3,5,7,4′‐tetrahydroxyflavone)), and isoflavones (prunetin (5,4′‐dihydroxy‐7‐methoxyisoflavone)). These differences in content may lead to variations in the antioxidant activity of *C. tangutica* at different altitudes.

#### 3.2.4 Amino Acids and Derivatives

A total of 204 amino acids and derivatives were detected, including 21 amino acids. Among these, 7 are essential for humans: lysine, tryptophan, phenylalanine, valine, isoleucine, leucine, methionine; the remaining 14 are non‐essential: cystine, glycine, citrulline, isoserine, homomethionine, pyroglutamic acid, norleucine, aspartic acid/asparagine, arginine, proline, histidine, tyrosine, glutamic acid/glutamine, ornithine. Among them, glutamic acid/glutamine and lysine have the highest content, while cystine has the lowest. Although the composition of amino acids and derivatives in samples from different altitudes is similar, their content varies significantly. The content of proline, tyrosine, lysine, glutamine, and others in the JZ is significantly lower than in other regions. The GH has significantly higher levels of amino acids and derivatives, such as citrulline, methionine, isoserine, Met‐Arg‐Ser, and Trp‐Arg‐His, compared to other regions. Consistent with the changes in flavonoid compound content, the levels of most amino acids and derivatives first increase and then decrease with increasing altitude, peaking in the GH with compounds like methionine, tryptophan, and His‐Tyr. Additionally, some amino acids and derivatives such as N‐acetyl‐L‐methionine and ornithine, increase with altitude, while others, like Arg‐Met‐Met, gradually decrease.

#### 3.2.5 Phenolic Acids

A total of 204 free and bound phenolic acids were detected, including free phenolic acids such as caffeic acid, chlorogenic acid, α‐hydroxycinnamic acid, 4‐hydroxybenzoic acid, ferulic acid, isoferulic acid, salicylic acid, homogentisic acid, syringic acid, gentisic acid, coumaric acid, and isovanillic acid. Additionally, three isomers of chlorogenic acid were detected: neochlorogenic acid, cryptochlorogenic acid, and isochlorogenic acid. The highest contents were found in caffeic acid, chlorogenic acid, α‐hydroxycinnamic acid, 4‐hydroxybenzoic acid, ferulic acid, isoferulic acid, and salicylic acid, while the lowest were in isovanillic acid and coumaric acid. Bound acids included protocatechuic acid, caffeic acid, 4‐hydroxybenzoic acid, salicylic acid, vanillic acid, sinapic acid, ferulic acid, coumaric acid, gallic acid, syringic acid, and gentisic acid. The most abundant were protocatechuic acid, vanillic acid, caffeic acid, coumaric acid, ferulic acid, chlorogenic acid, syringic acid, gallic acid, and salicylic acid, while the least abundant was sinapic acid. Additionally, phenolic acids unique to medicinal plants, such as trollioside (Ming et al. 2016), scroside (Wang et al. 2004), and salidroside (Zhang, Xie, et al. 2021) were detected, which possess anti‐inflammatory, anti‐oxidative stress, lipid‐lowering, and anti‐apoptotic functions.

The phenolic acid content varies significantly among samples from different altitudes. Syringic acid content is significantly higher in DQ and ZD regions compared to other regions, and α‐hydroxycinnamic acid and 4‐hydroxybenzoic acid content is significantly higher in the ZD than in other regions. In the JZ region, chlorogenic acid, caffeic acid, salicylic acid, and coumaric acid content in *C. tangutica* is significantly higher than in other regions. However, protocatechuic acid and gentisic acid content is significantly lower than in other groups, with their content increasing with altitude. Ferulic acid and isoferulic acid content in the GH region is significantly higher than in other regions, whereas isovanillic acid content is significantly lower. Homogentisic acid content is significantly higher in MY, GH, and DQ regions compared to JZ and ZD regions.

#### 3.2.6 Alkaloids

A total of 122 alkaloid metabolites were detected, including 49 alkaloids, 24 plumeranes, 16 phenolamines, 10 pyridine alkaloids, 7 quinoline alkaloids, and 6 isoquinoline alkaloids. Among these, quinoline alkaloids (xanthurenic acid 8‐O‐glucoside, 3‐quinolinecarboxylic acid, 8‐hydroxyquinoline) had the highest content, while quinine had the lowest content and was not identified in the MY and DQ regions. Significant differences in the alkaloid content were observed among samples from different altitudes. In the MY, GH, and DQ regions, most significantly different alkaloid metabolites exhibited higher contents compared to other regions. In contrast, the JZ had significantly lower alkaloid contents, followed by the ZD.

#### 3.2.7 Terpenoids

A total of 56 terpenoid metabolites were detected, comprising 20 monoterpenoids, 16 sesquiterpenoids, 8 diterpenoids, 7 triterpenes, and 5 triterpene saponins. Among these, sesquiterpenoids (6β‐hydroxy‐8α‐methoxyeremophila‐1(10),7(11)‐dien‐12,8β‐olide) had the highest content, while monoterpenoids (catalpol and secoxyloganin) exhibited the lowest content. Most monoterpenoids showed higher contents in the JZ compared to the other regions, particularly bartsioside, sweroside, monotropein, and dehydroxysecologanic acid, which were significantly elevated. Most sesquiterpenoids exhibited significantly higher contents in the GH relative to other regions. Diterpenoids (such as levopimaric acid, isopimaric acid, kaurenoic acid, pimaric acid) exhibited significantly higher contents in the JZ compared to other regions. Most triterpenes increased gradually with altitude. Five triterpene saponins (hederagenin‐3‐O‐[β‐D‐glucosyl‐(1–3)‐a‐L‐rhamnosyl‐(1–2)‐a‐L‐arabinoside], pulchinenoside C, pulsatilla saponin D, hederacoside C, hederacoside D) exhibited significantly higher contents in the MY, GH, and ZD compared to the ZJ.

## 4 Discussion

### 4.1 MaxEnt Models Predicted Changes in the Potential Habitat and Area of *C. tangutica* on the Qinghai Plateau

The Qinghai Plateau, situated in the northeastern part of the Tibetan Plateau, is rich in plant species. However, significant geographical and climatic variations result in distinct regional differences in these species (Mao et al. 2021). The southeastern region of the plateau features a relatively mild climate and abundant plant species, whereas the northwest is characterized by extreme aridity, numerous glaciers, and rocky terrain, resulting in a sharp decrease in plant diversity (Wang et al. 2012). Global climate warming is causing the habitats of some species to expand, shift, or contract, leading to changes in species richness (Dirnböck et al. 2011; Li et al. 2015; Maclean and Early 2023). Therefore, predicting the distribution of potential suitable habitats for plants and their migration trends under future climate change can provide scientific support for the ecological conservation, management, and utilization of plants (Shi et al. 2023). In this study, we employed the MaxEnt model for species distribution modeling (SDM) to predict the potential distribution of *C. tangutica* on the Qinghai Plateau and explored the potential impacts of climate change on its habitat distribution. The results indicate that, under current climate conditions, the high and moderate suitable habitats of *C. tangutica* on the Qinghai Plateau are primarily located in the eastern, central‐northern, and southern regions, covering an area of 299,300 km^2^. These areas are characterized by altitudes ranging from 2000 to 4000 m, relatively mild climates, and dominance of grasslands and shrubs. In contrast, the distribution of *C. tangutica* is lower in the extreme climates of the northwest saline‐alkali lands and the southwestern plateau hinterland, highlighting the close relationship between its habitat distribution and the plateau's environmental conditions.

With rising annual greenhouse gas emissions, global temperature increases seem inevitable (Singh et al. 2023). To simulate the potential impact of global temperature rise on the habitat of *C. tangutica* , we employed the MaxEnt model to predict habitat area changes under four greenhouse gas emission pathways from CMIP6 models. The results indicate that, under future climate change, the area and distribution of high, moderate, and low suitable habitats for *C. tangutica* will change to varying degrees. Specifically, the area of high suitable habitat increases by 3.89% only under the SSP5‐8.5 pathway in the 2050s, primarily in the southeastern Guoluo region (average altitude above 4000 m) and high‐latitude northern and northeastern regions. This is consistent with previous studies indicating a shift in potential suitable habitat distribution toward higher altitudes and latitudes due to climate change (Shi et al. 2023). Other climate scenarios show decreases and fragmented distributions, mainly in the eastern and northwestern regions. The area of moderate suitable habitat increases under the SSP2‐4.5 pathway, with regions that were previously high suitable becoming moderate, and formerly low suitable areas also becoming moderate. In contrast, decreases occur under the SSP5‐8.5 pathway, particularly in the central‐northern and northwestern regions. Furthermore, compared to current climate conditions, the total suitable habitat area for *C. tangutica* decreases under all future climate scenarios, with more drastic reductions under the SSP5‐8.5 pathway, indicating a greater reduction in suitable habitat area with increasing radiative forcing. These findings provide valuable information and reasonable references for the future development and conservation of wild *C. tangutica* .

### 4.2 The Primary Bioclimatic Variables Influencing the Distribution of *C. tangutica* on the Qinghai Plateau Have Been Identified

Studies indicate that the potential distribution of species is influenced by variables such as temperature, precipitation, topography, soil, UVB, demographic characteristics, and other environmental factors (Thammanu et al. 2021). In this study, we evaluated the potential habitat distribution and the main environmental variables affecting *C. tangutica* using an optimized MaxEnt model, based on 130 valid distribution records and 46 collected environmental variables. Based on the percentage contribution and jackknife test of the MaxEnt model, we identified UV‐B seasonality (uvb2) and elevation (elev) as the main environmental variables influencing the potential habitat distribution of *C. tangutica* . UVB and elevation were found to have the most significant impact, while temperature and soil factors had minimal influence on the distribution of *C. tangutica* . The species predominantly inhabits high‐altitude plateau grasslands, shrubs, or roadsides, characterized by strong UV radiation, high altitude, and low precipitation (Guo, Wang, et al. 2023), confirming consistency between our predictions and the preferred habitat conditions of *C. tangutica* . To further clarify the biological tolerance and habitat preferences of *C. tangutica* , we analyzed the response curves of the two main environmental variables that significantly affect its distribution. We obtained critical values for uvb2 between 239,062.77 J·m^−2^·d^−1^ and 268,462.59 J·m^−2^·d^−1^, and for elev between 2801.29 and 4292.36 m (existence probability > 0.5), indicating the highest habitat suitability for *C. tangutica* within these ranges. In our comprehensive survey of *C. tangutica* natural resources across the region, we found that the species is most commonly distributed in areas with an average altitude ranging from 2500 to 4000 m, consistent with our predictions.

### 4.3 Metabolomic Identification of Major Metabolites and Their Contents in *C. tangutica* Leaves Indicates That Altitude May Cause Variations in Metabolite Content

Current research on *C. tangutica* mainly focuses on chloroplast genome assembly analysis (Guo et al. 2024), flower color variation (Guo, Wang, et al. 2023), flower traits, sexual distribution (Zhao et al. 2023), and functional activities of major triterpenoid saponins (Wei et al. 2023b; Zhao et al. 2016; Zhong et al. 2001). However, other functional components have not been thoroughly studied, thereby limiting their utilization. In this study, based on the results predicted by the MaxEnt model and the primary environmental variables influencing the distribution of *C. tangutica* , we conducted a comprehensive metabolomic analysis of *C. tangutica* leaves from five different regions. Metabolomic analysis identified a total of 1362 compounds, including 255 flavonoids, 204 phenolic acids, 122 alkaloids, 56 terpenoids, and other metabolites. Some compounds known for their anti‐inflammatory, antioxidant, anti‐tumor, antibacterial, antiviral, and neuroprotective effects were found to accumulate significantly in *C. tangutica* . These include phenolic acids such as chlorogenic acid, caffeic acid, ferulic acid, isoferulic acid, and salicylic acid (Rashmi and Negi 2020; Yang, Lan, and Sun 2023), flavonoids like flavonols, flavones, and isoflavones (Panche et al. 2016), alkaloids including indole alkaloids, phenylethylamines, pyridine alkaloids, and quinoline alkaloids (Ziegler and Facchini 2008), and terpenoids such as diterpenes, sesquiterpenes, triterpenes, triterpenoid saponins (Pichersky and Raguso 2018; Yang et al. 2020). Additionally, nine quinone compounds with anti‐inflammatory, antimicrobial, insecticidal, and hypotensive effects, as well as three tannin compounds, were identified (Das et al. 2020; Lu et al. 2013).

In this study, based on the MaxEnt model prediction, the highest suitability altitude range for *C. tangutica* was determined to be between 3095.09 m and 3300.76 m (existence probability > 0.62). GH (elev = 3191 m) was selected as the core group, and differential metabolite analysis was conducted compared to other regions (JZ/MY/DQ/ZD). A total of 1172 differential metabolites were identified, comprising 379, 334, 239, and 207 up‐regulated substances, and 268, 284, 366, and 406 down‐regulated substances in the four comparison groups, respectively. This suggests that as altitude increases, the number of up‐regulated differential metabolites decreases, while down‐regulated metabolites increase, indicating significant differences in metabolite content in *C. tangutica* leaves at different altitudes. Comparative analysis revealed significant variations in the content of antioxidant‐active substances at different altitudes, potentially leading to differences in anti‐inflammatory, antioxidant, antibacterial, and antiviral functions in *C. tangutica* (Jugran et al. 2016). Studies by Nasir Khan et al. indicated that plants from higher altitudes, due to their higher levels of secondary metabolites, are more suitable as medicinal herbs compared to those from lower altitudes (Khan et al. 2016). Our study found that most flavonoids, amino acids and derivatives, and alkaloids in *C. tangutica* showed an initial increase followed by a decrease with increasing altitude, with relatively higher levels observed in MY (2641 m), GH (3191 m), and DQ (3590 m), and lower levels in JZ (2060 m) and ZD (4252 m). This suggests that *C. tangutica* accumulates more secondary metabolites and thus has higher medicinal value in the altitude range of 2500 to 3500 m. Additionally, triterpenoid saponins, the major bioactive compounds in *C. tangutica* , were found to accumulate significantly higher in MY, GH, and ZD compared to ZJ. This indicates that the high accumulation of triterpenoid saponins helps *C. tangutica* adapt faster to high‐altitude environmental conditions (Hashim et al. 2020). However, we also observed significantly lower levels of triterpenoid saponins in DQ compared to MY, GH, and ZD, possibly because DQ is in the middle suitability zone where most plants generally accumulate fewer primary chemical constituents (Wan et al. 2021). The habitat suitability changes and altitude effects on the enrichment of functional active compounds revealed in this study provide a scientific basis for selecting and cultivating medicinal resources. Notably, samples from highly suitable areas show higher levels of pharmacologically active substances, such as antioxidants and antibacterial compounds, identifying potential regions for further pharmacological research and compound isolation. Considering both medicinal and ecological value, conservation of highly suitable areas in the eastern and central regions is recommended through the establishment of native germplasm conservation zones. In areas at elevations of 2500 to 3500 m, semi‐artificial propagation and community co‐management models could balance the medicinal needs of mountain residents with the long‐term sustainability of the species. Moreover, dynamic monitoring and policy support should be enhanced to address future habitat suitability shifts and prevent resource decline from overharvesting.

## 5 Conclusions

In this study, we combined the MaxEnt model with ultra‐performance liquid chromatography–tandem mass spectrometry (UPLC‐MS/MS) for the first time to predict the habitat suitability of *C. tangutica* on the Qinghai Plateau and to analyze variations in major leaf metabolites across different altitudes. The results indicated that UV‐B seasonality (uvb2) and altitude were the primary environmental variables influencing the distribution of *C. tangutica* . It is primarily distributed in eastern, central, and parts of southern Qinghai, with high‐ and medium‐suitability areas spanning approximately 299,300 km^2^. Under future climate change, the suitable habitat range of *C. tangutica* is projected to shrink, with total suitable areas decreasing by 10.93% in the 2050s and 6.77% in the 2070s under the SSP5‐8.5 scenario, while shifting toward higher altitudes and latitudes. A total of 1362 metabolites were identified in the leaves of *C. tangutica* , including 255 flavonoids, 204 amino acids and derivatives, 204 phenolic acids, 122 alkaloids, and 56 terpenoids, among others. The contents of major metabolites in the leaves varied across different altitudes. Triterpenoid saponin levels, which exhibit antioxidant and antibacterial activities, were significantly higher at elevations of 2500–3500 m compared with regions below 2000 m or above 4000 m. These findings fill a research gap regarding the ecological adaptability of *C. tangutica* and provide a theoretical basis for the future sustainable utilization of its medicinal and ornamental value.

## Author Contributions

**Haiwang Zhang:** formal analysis (equal), investigation (equal), software (equal), writing – original draft (equal). **Xiaozhu Guo:** formal analysis (equal), investigation (equal), resources (equal). **Xiaoqiang Wei:** formal analysis (equal), resources (equal). **Lihui Wang:** writing – review and editing (equal). **Qiwen Zhong:** writing – review and editing (equal). **Xuemei Sun:** resources (equal), writing – review and editing (equal).

## Disclosure

*Statement on permissions and voucher specimens*: Permissions or licenses for collecting *C. tangutica* were obtained prior to the study. Voucher specimens have been deposited in a public herbarium, ensuring access to the deposited material.

*Identification and voucher specimen information*: The formal identification of the plant material used in this study was undertaken by Northwest Institute of Plateau Biology. A voucher specimen has been deposited in the Herbarium, Northwest Institute of Plateau Biology, CAS, and the deposition number is HNWP 0208921. This ensures the material is publicly accessible for future reference.

## Conflicts of Interest

The authors declare no conflicts of interest.

## Supporting information


**Data S1:** ece372110‐sup‐0001‐Supinfo01.zip.

## Data Availability

The data that support the findings of this study are available in the Supporting Information S1 of this article.
