# Supplementary material for: Potential Distribution Prediction and Metabolite Analysis of Clematis tangutica (Maxim.) Korsh. On the Qinghai Plateau
Source: Ecol Evol. 2025 Sep 30;15(10):e72110. doi: 10.1002/ece3.72110 (PMC12483990; doi:10.1002/ece3.72110)
Supplement: Supplementary file 1 — Data S1: ece372110‐sup‐0001‐Supinfo01.zip. [file ECE3-15-e72110-s001.zip › Table S1-S2.docx]

Table S1 Environmental variables and running environmental variables.

| Code | Environment variables |
| --- | --- |
| **bio1** | **Annual Mean Temperature/℃** |
| bio2 | Mean Diurnal Range (Mean of monthly (max temp - min temp))/℃ |
| bio3 | Isothermality (BIO2/BIO7) (×100) |
| **bio4** | **Temperature Seasonality (standard deviation ×100)** |
| bio5 | Max Temperature of Warmest Month/℃ |
| bio6 | Min Temperature of Coldest Month/℃ |
| bio7 | Temperature Annual Range (BIO5-BIO6) |
| **bio8** | **Mean Temperature of Wettest Quarter/℃** |
| **bio9** | **Mean Temperature of Driest Quarter/℃** |
| bio10 | Mean Temperature of Warmest Quarter/℃ |
| **bio11** | **Mean Temperature of Coldest Quarter/℃** |
| bio12 | Annual Precipitation/mm |
| bio13 | Precipitation of Wettest Month/mm |
| bio14 | Precipitation of Driest Month/mm |
| bio15 | Precipitation Seasonality (Coefficient of Variation) |
| bio16 | Precipitation of Wettest Quarter/mm |
| **bio17** | **Precipitation of Driest Quarter/mm** |
| **bio18** | **Precipitation of Warmest Quarter/mm** |
| **bio19** | **Precipitation of Coldest Quarter/mm** |
| **elevation** | **elevation/m** |
| slope | slope/° |
| aspect | aspect/rad |
| awc | Topsoil Available water storage capacity |
| t_texture | Topsoil Texture |
| t_gravel | Topsoil Gravel Content |
| t_sand | Topsoil Sand Fraction |
| t_silt | Topsoil Silt Fraction |
| **t_clay** | **Topsoil Clay Fraction** |
| **t_usda_tex** | **Topsoil USDA Texture Classification** |
| t_ref_bulk | Topsoil Reference Bulk Density |
| t_oc | Topsoil Organic Carbon |
| t_pH | Topsoil pH(H_2_O) |
| t_cec_clay | Topsoil CEC(clay) |
| t_cec_soil | Topsoil CEC(soil) |
| **t_bs** | **Topsoil Base Saturation** |
| t_teb | Topsoil TEB |
| t_caco_3_ | Topsoil Calcium Carbonate |
| t_caco_4_ | Topsoil Gypsum |
| **t_esp** | **Topsoil Sodicity(ESP)** |
| t_ece | Topsoil Salinity(Elco) |
| **uvb1** | **Annual Mean UV-B** |
| **uvb2** | **UV-B Seasonality** |
| **uvb3** | **Mean UV-B of Highest Month** |
| **uvb4** | **Mean UV-B of Lowest Month** |
| **uvb5** | **Sum of Monthly Mean UV-B during Highest Quarter** |
| uvb6 | Sum of Monthly Mean UV-B during Lowest Quarter |

**Note:** The bolded parts are the actual running environment variables

Table S2 Changes in the area of suitable habitat(‘-’Indicates a reduction).

| Periods | Routing Pattern | High Potential | | Medium Potential | | Low Potential | | Total Potential | |
| --- | --- | --- | --- | --- | --- | --- | --- | --- | --- |
|  |  | Area（10^4^km^2^） | Increase(%) | Area（10^4^km^2^） | Increase(%) | Area（10^4^km^2^） | Increase(%) | Area（10^4^km^2^） | Increase(%) |
| Current |  | 14.50 |  | 15.43 |  | 18.37 |  | 48.29 |  |
| 2050s | SSP2-4.5 | 14.13 | -2.51% | 16.20 | 4.99% | 16.12 | -12.21% | 46.45 | -3.81% |
|  | SSP5-8.5 | 15.06 | 3.89% | 12.85 | -16.71% | 15.10 | -17.77% | 43.01 | -10.93% |
| 2070s | SSP2-4.5 | 11.74 | -19.02% | 17.50 | 13.44% | 17.82 | 2.99% | 47.06 | -2.55% |
|  | SSP5-8.5 | 13.98 | -3.55% | 13.85 | -10.24% | 17.19 | -6.41 | 45.02 | -6.77% |
