# Supplementary material for: Potential Distribution Prediction and Metabolite Analysis of Clematis tangutica (Maxim.) Korsh. On the Qinghai Plateau
Source: Ecol Evol. 2025 Sep 30;15(10):e72110. doi: 10.1002/ece3.72110 (PMC12483990; doi:10.1002/ece3.72110)

Fig. S1 Correlation analysis of environmental variable values at the occurrence points of *C. tangutica*: (a) climatic and topographic variables; (b) soil and UV variables.


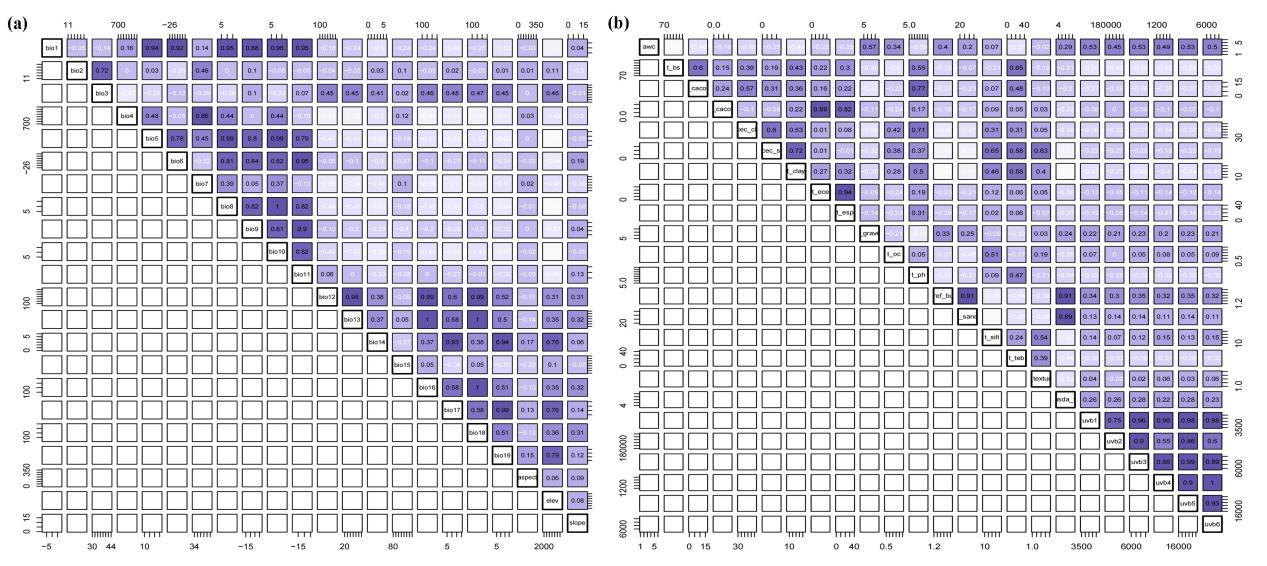


Fig. S2 Optimization of the MaxEnt model and ROC curve results. (a) AICc values of the MaxEnt model generated by ENMeval. Smaller AICc values indicate better model fit. (b) Mean values of AUC for training and testing. Larger AUC values indicate higher accuracy in model simulation.


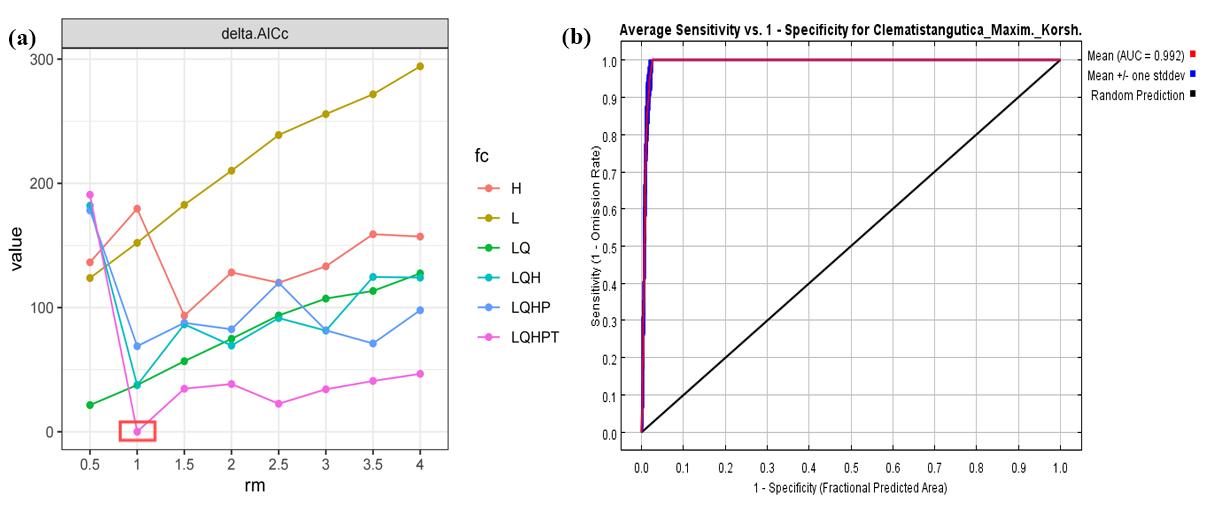


Fig. S3 Jackknife of regularized training gain for *C. tangutica* of environmental variables distribution.


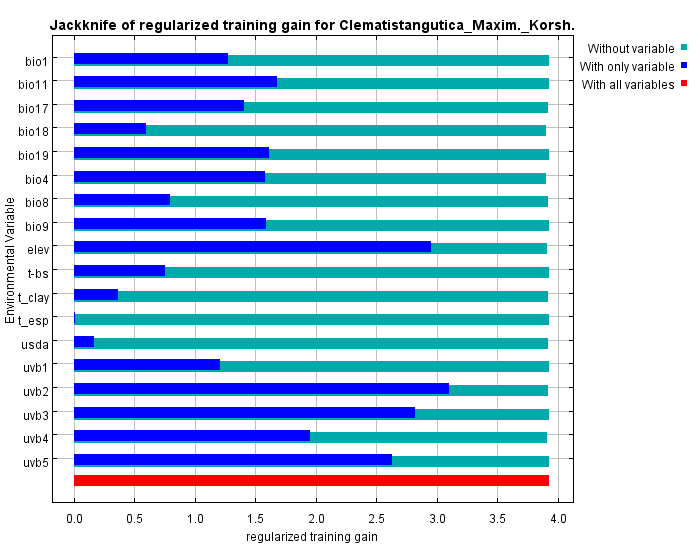


Fig.S4 The OPLS-DA score plot and S-plot. (a-d) The OPLS-DA score plots comparing: GH vs. JZ, GH vs. MY, GH vs. DQ, GH vs. ZD. (e-h) The S-plot plots comparing: GH vs. JZ, GH vs. MY, GH vs. DQ, GH vs. ZD.


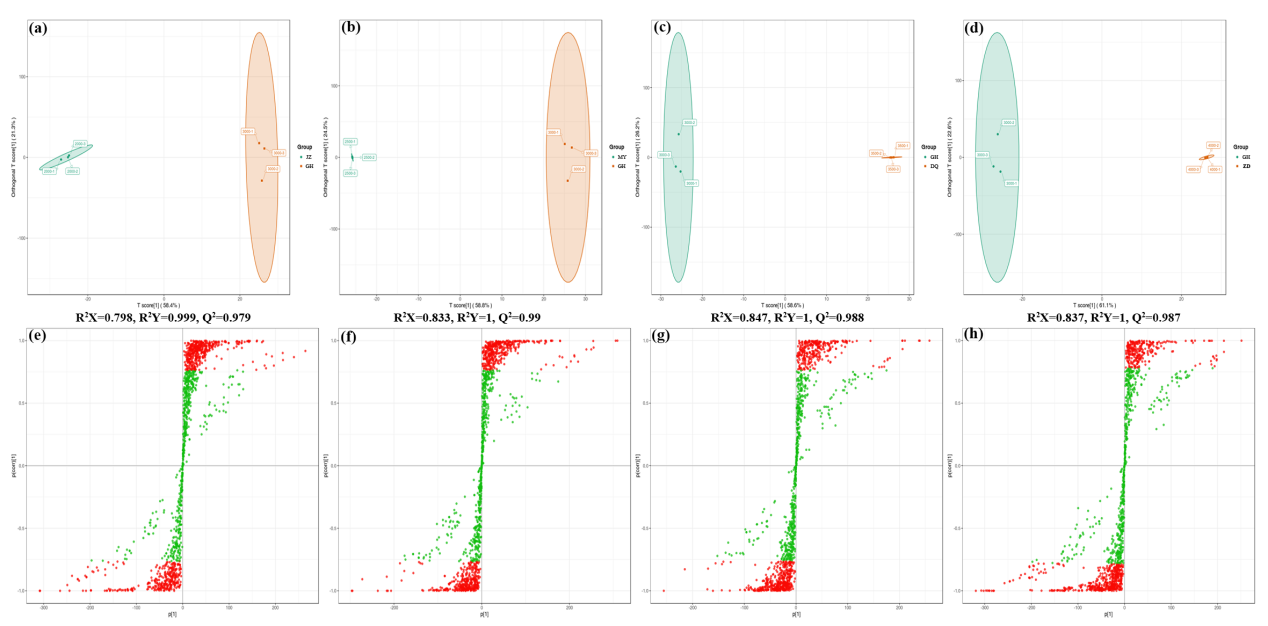

Supplement: Supplementary file 1 — Data S1: ece372110‐sup‐0001‐Supinfo01.zip. [file ECE3-15-e72110-s001.zip › Fig S1-S4.docx]
