# Supplementary figures and images for: Potential Distribution Prediction and Metabolite Analysis of Clematis tangutica (Maxim.) Korsh. On the Qinghai Plateau
Source: Ecol Evol. 2025 Sep 30;15(10):e72110. doi: 10.1002/ece3.72110 (PMC12483990; doi:10.1002/ece3.72110)

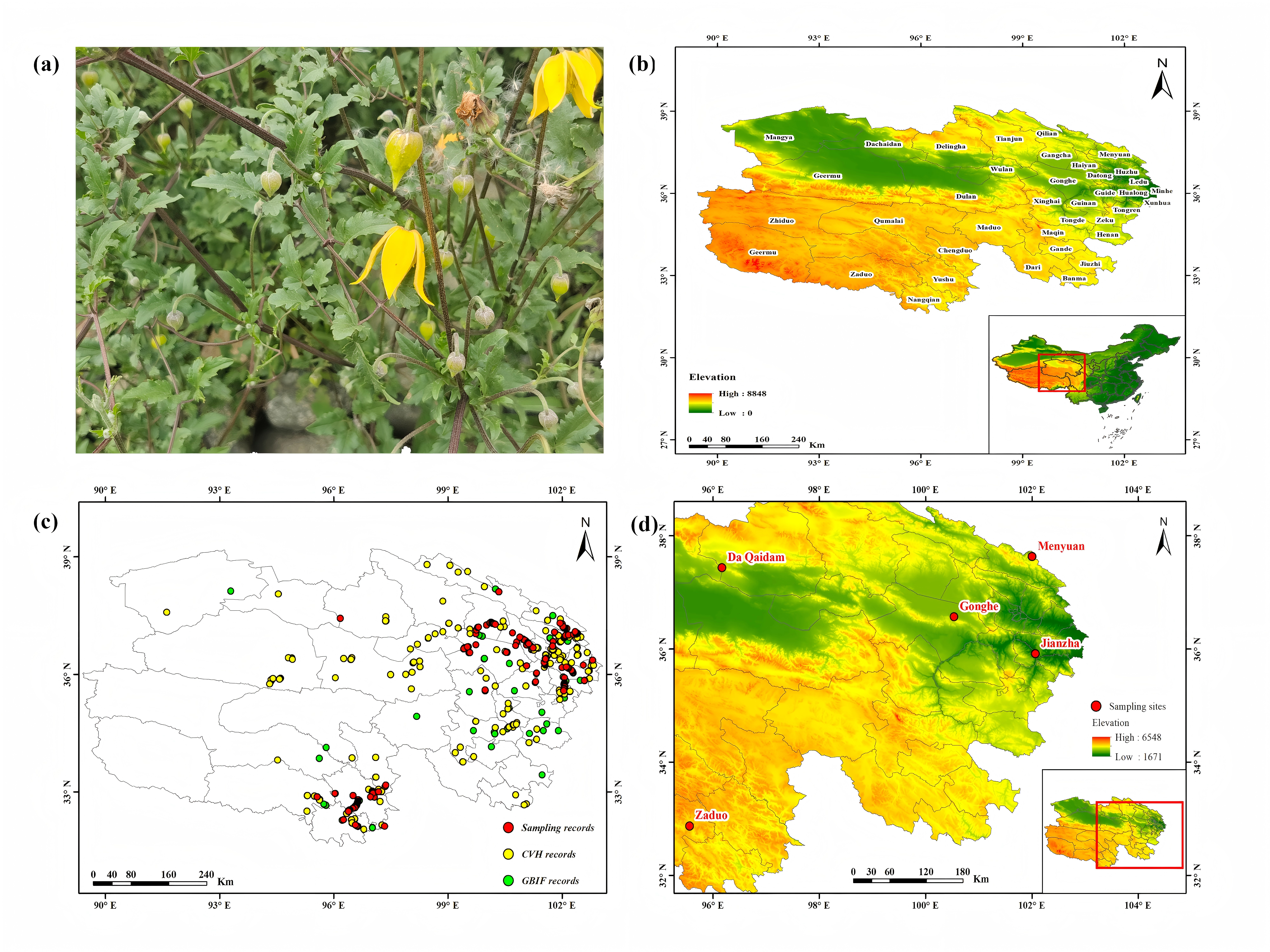

Supplement: Supplementary file 1 — Data S1: ece372110‐sup‐0001‐Supinfo01.zip. [file ECE3-15-e72110-s001.zip › Fig. 1.png]

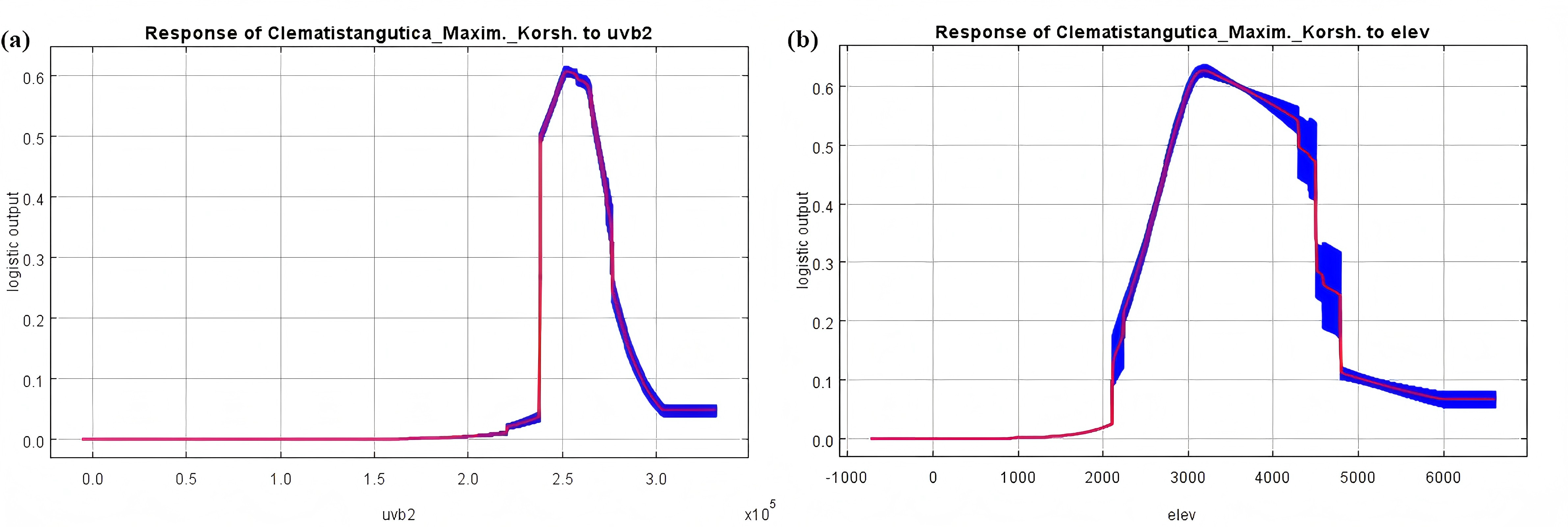

Supplement: Supplementary file 1 — Data S1: ece372110‐sup‐0001‐Supinfo01.zip. [file ECE3-15-e72110-s001.zip › Fig. 2.png]

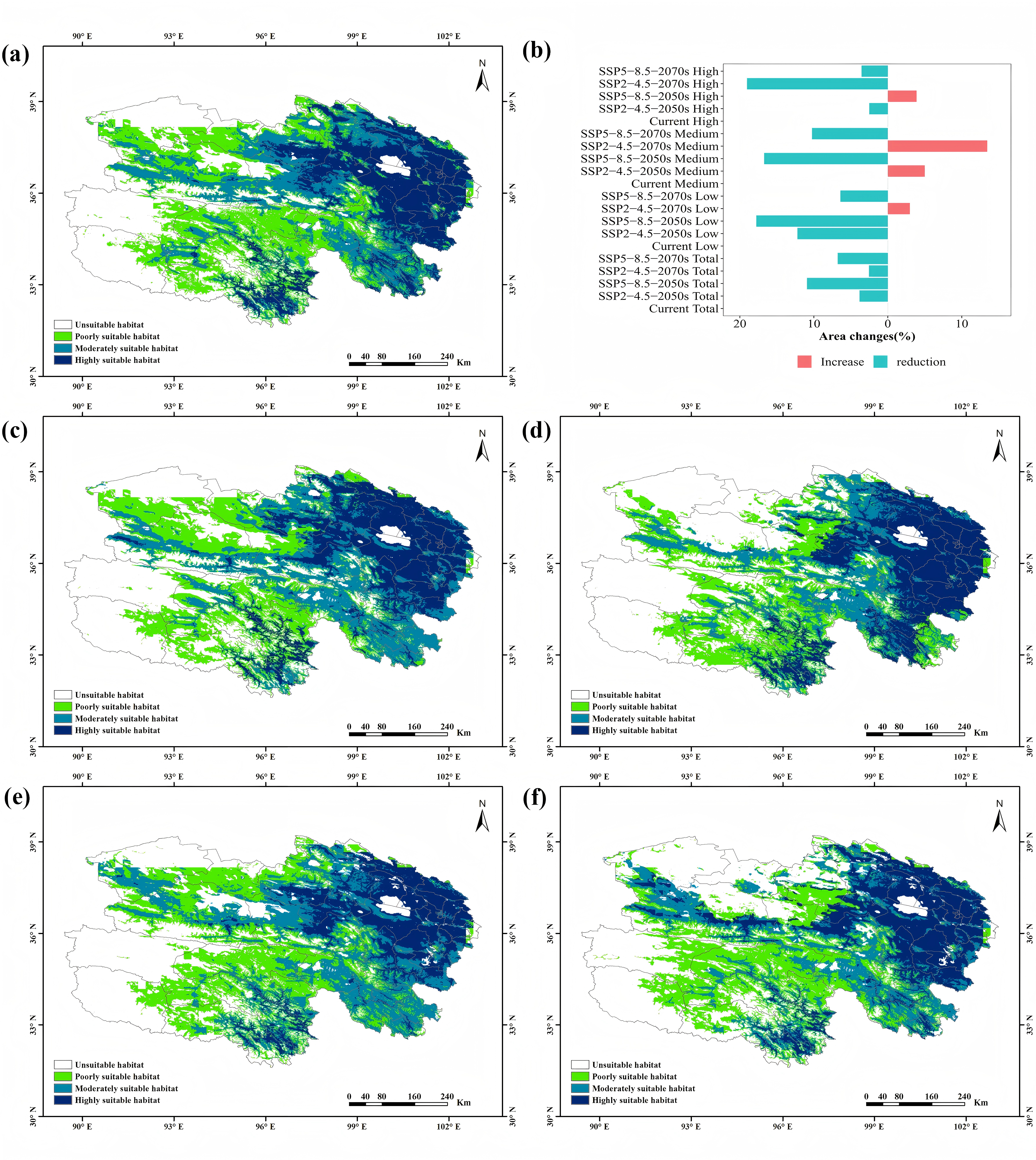

Supplement: Supplementary file 1 — Data S1: ece372110‐sup‐0001‐Supinfo01.zip. [file ECE3-15-e72110-s001.zip › Fig. 3.png]

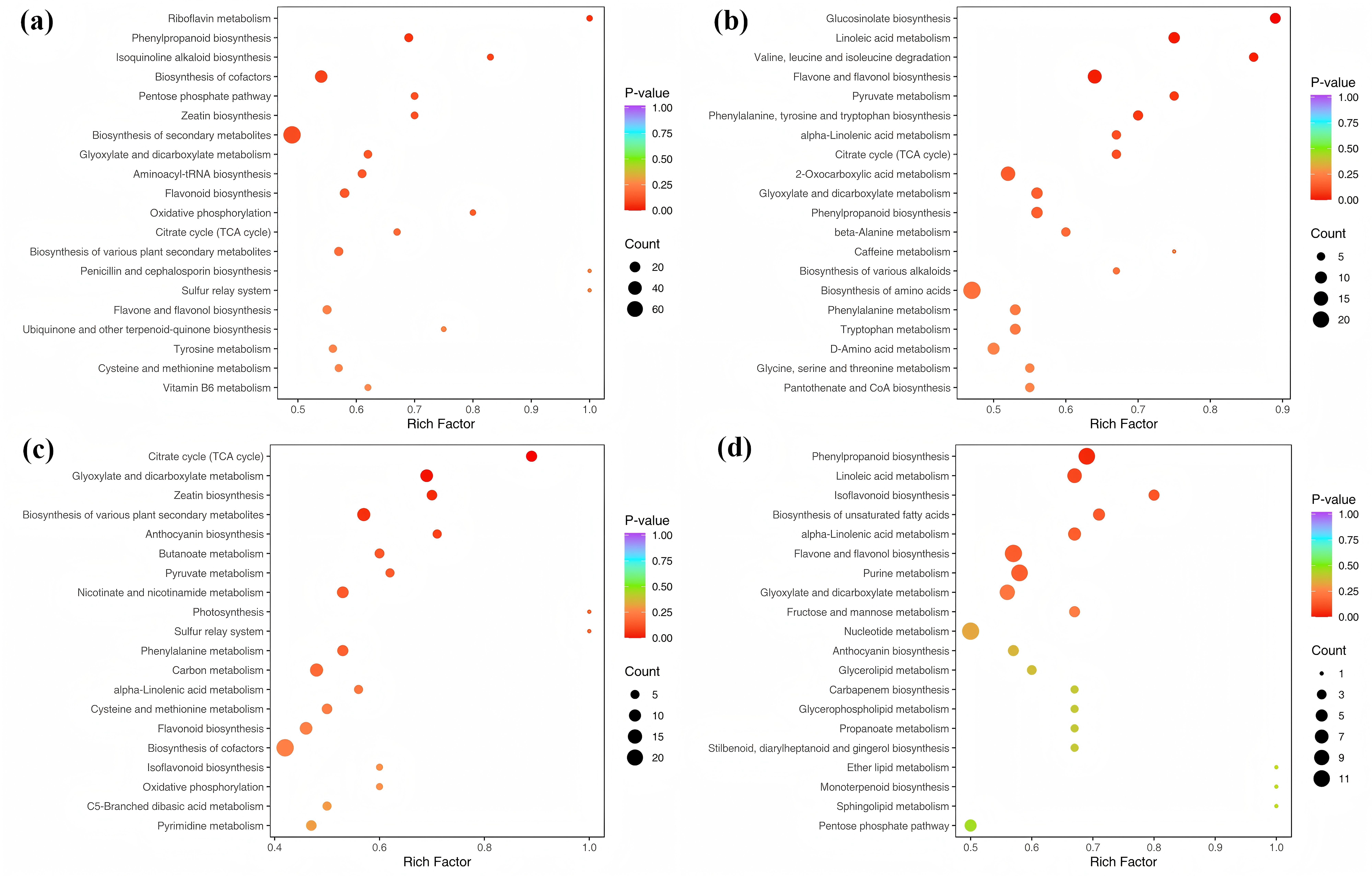

Supplement: Supplementary file 1 — Data S1: ece372110‐sup‐0001‐Supinfo01.zip. [file ECE3-15-e72110-s001.zip › Fig. 5.png]

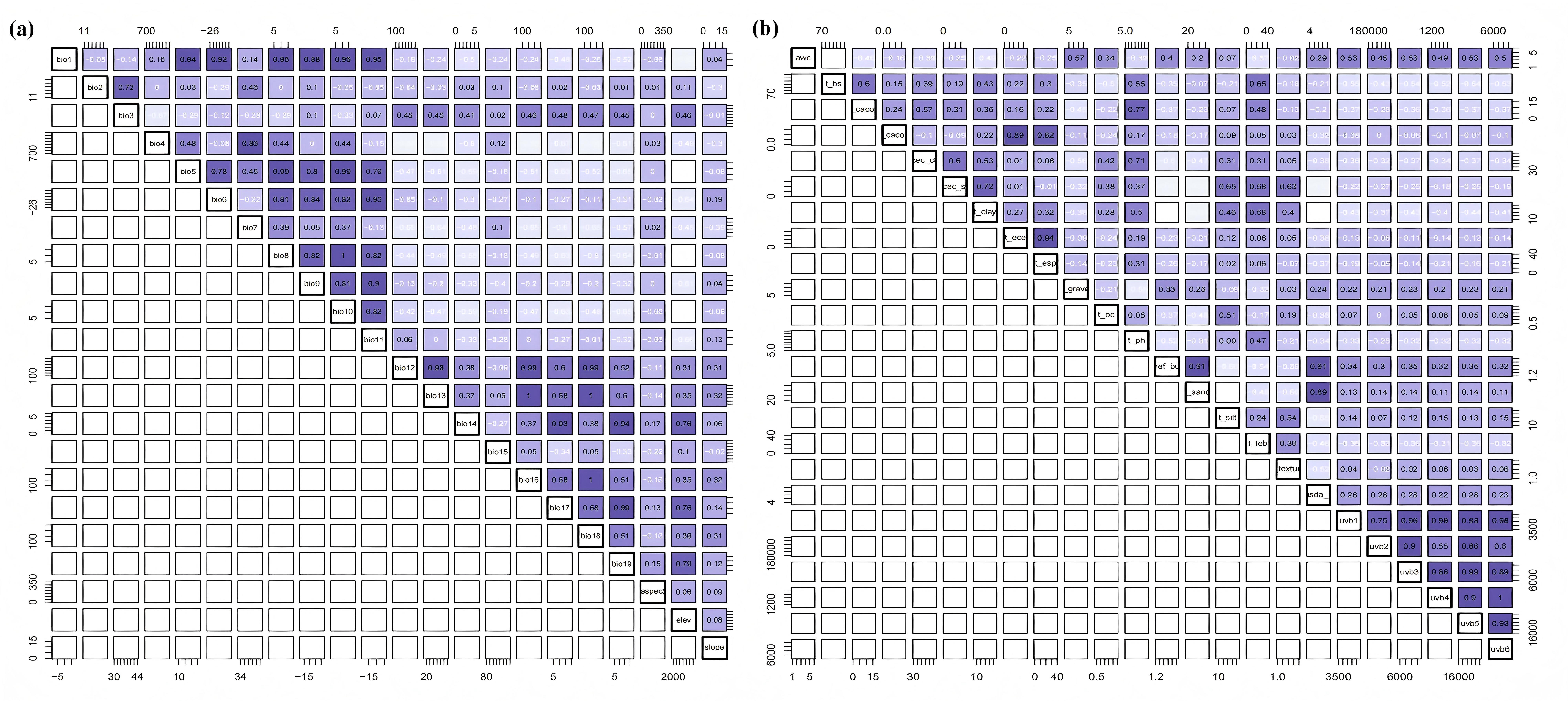

Supplement: Supplementary file 1 — Data S1: ece372110‐sup‐0001‐Supinfo01.zip. [file ECE3-15-e72110-s001.zip › Fig. S1.png]

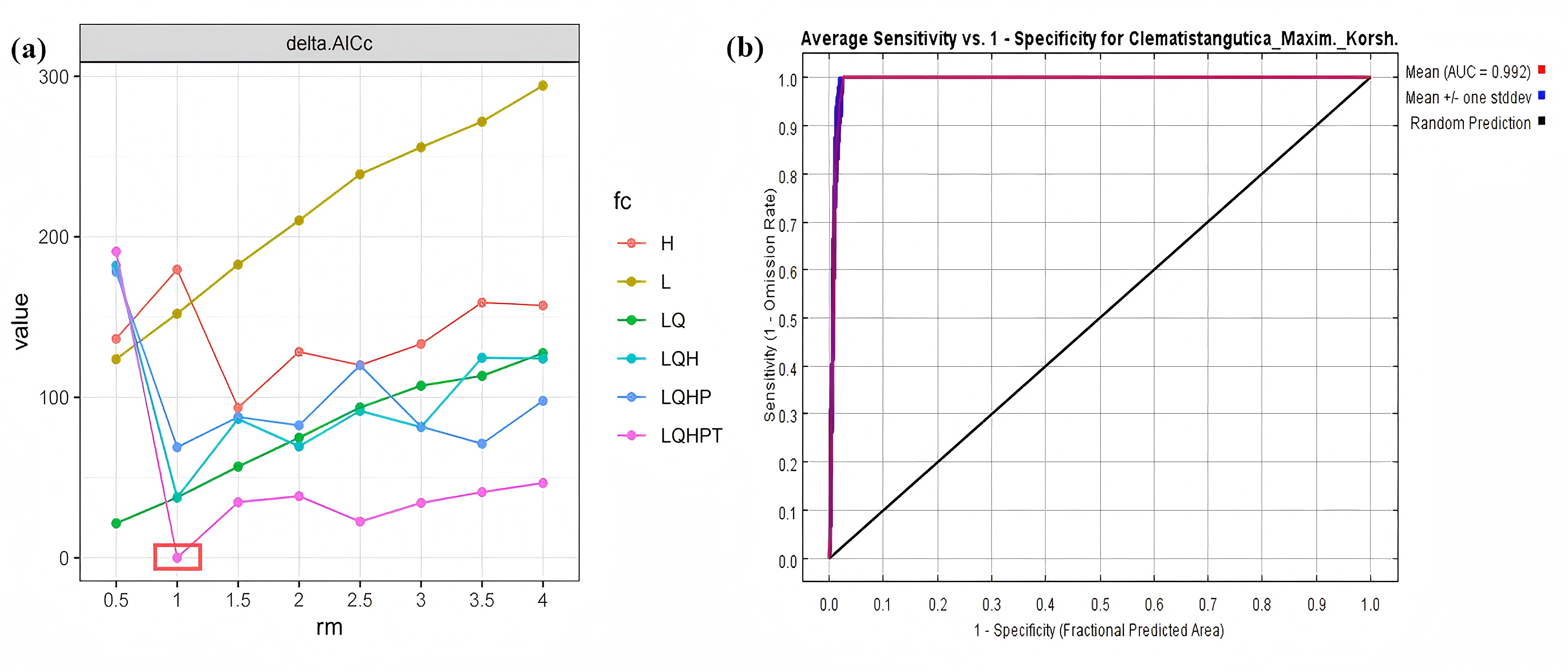

Supplement: Supplementary file 1 — Data S1: ece372110‐sup‐0001‐Supinfo01.zip. [file ECE3-15-e72110-s001.zip › Fig. S2.png]

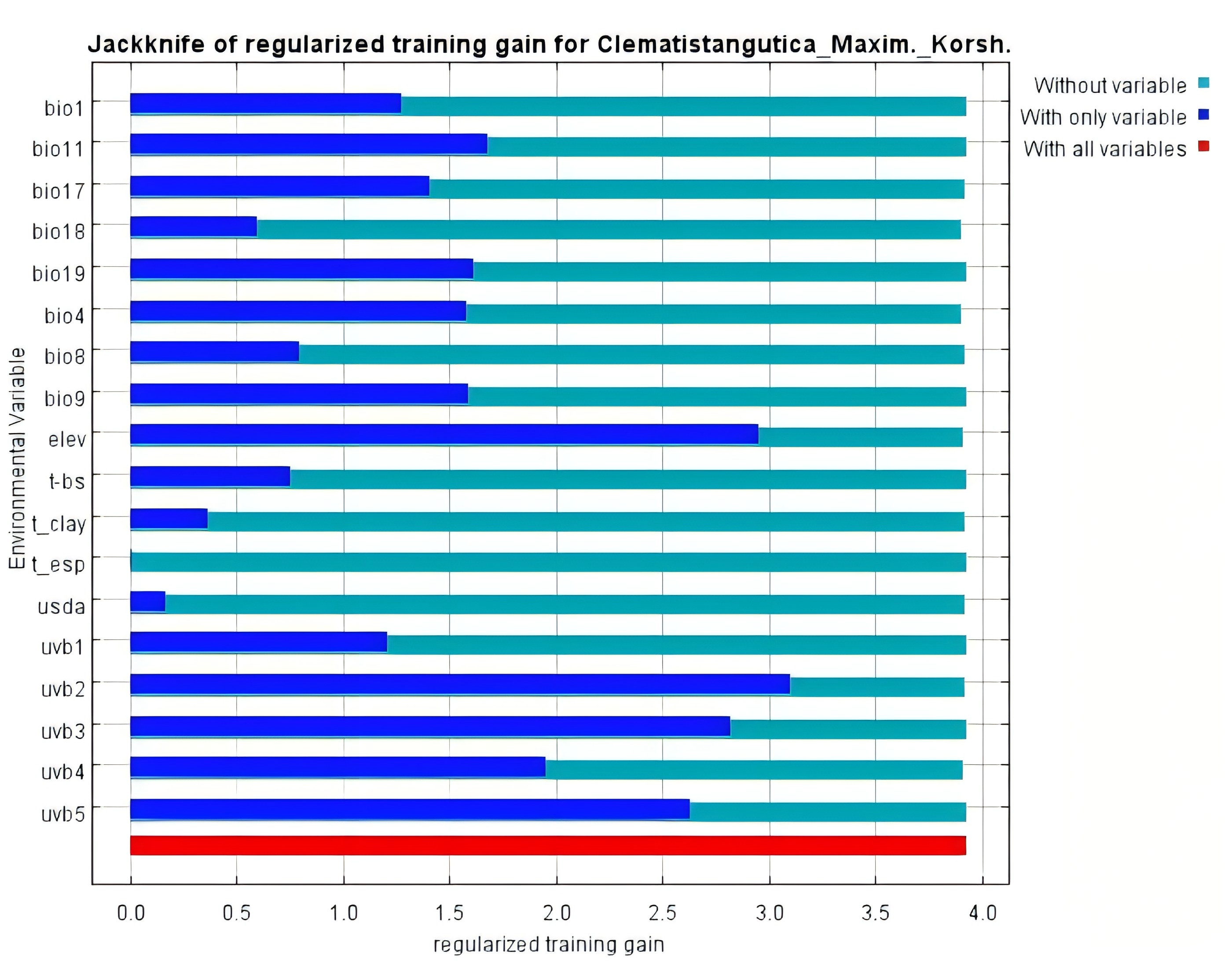

Supplement: Supplementary file 1 — Data S1: ece372110‐sup‐0001‐Supinfo01.zip. [file ECE3-15-e72110-s001.zip › Fig. S3.png]

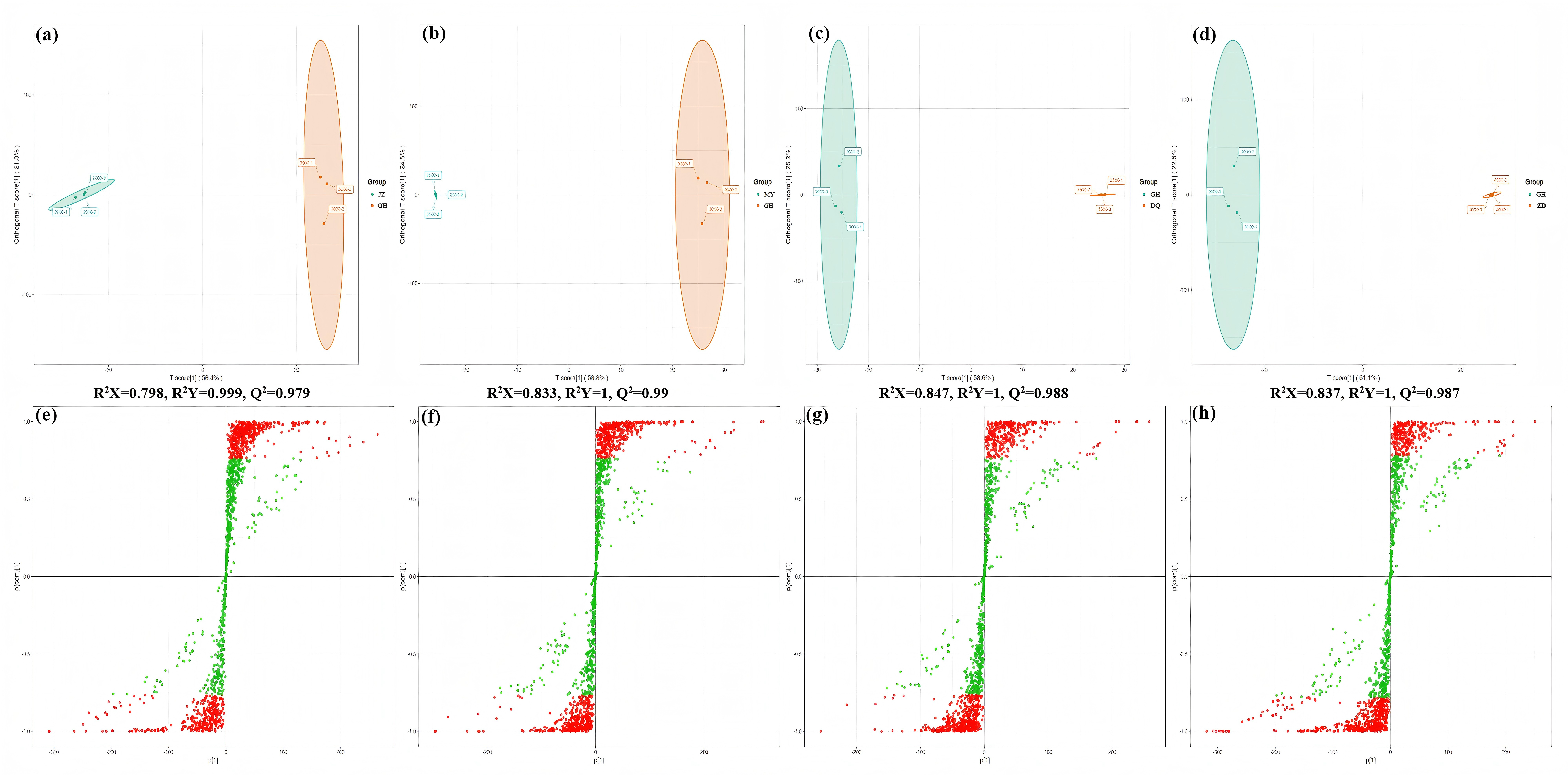

Supplement: Supplementary file 1 — Data S1: ece372110‐sup‐0001‐Supinfo01.zip. [file ECE3-15-e72110-s001.zip › Fig. S4.png]
